# Toward the synthesis, fluorination and application of N–graphyne†

**DOI:** 10.1039/d0ra08143d

**Published:** 2020-11-03

**Authors:** Gisya Abdi, Anna Filip, Michał Krajewski, Krzysztof Kazimierczuk, Marcin Strawski, Paweł Szarek, Bartosz Hamankiewicz, Zoran Mazej, Grzegorz Cichowicz, Piotr J. Leszczyński, Karol J. Fijałkowski, Andrzej Szczurek

**Affiliations:** Centre of New Technologies, University of Warsaw Żwirki i Wigury 93 02-097 Warsaw Poland a.szczurek@cent.uw.edu.pl; Laboratory of Molecular Medical Biochemistry, Nencki Institute of Experimental Biology, PAS 3 Pasteur Street 02-093 Warsaw Poland; Faculty of Chemistry, University of Warsaw Pasteura 1 02-093 Warsaw Poland; Department of Inorganic Chemistry and Technology, Jozef Stefan Institute Jamova cesta 39 SI 1000 Ljubljana Slovenia; The Czochralski Laboratory of Advanced Crystal Engineering, Faculty of Chemistry, University of Warsaw Żwirki i Wigury 101 02 089 Warsaw Poland

## Abstract

The discovery of properties and applications of unknown materials is one of the hottest research areas in materials science. In this work, we navigate a route towards these goals by the development of a new type of graphyne nanostructure. It is synthesised by a Sonogashira cross-coupling reaction of 1,3,5-triethynylbenzene with cyanuric chloride resulting in an extended carbon-based material called TCC. Also, we modify the obtained TCC *via* fluorination using XeF_2_ at various concentrations to investigate the effect of fluorination on the triple bonds and the conjugated structure of graphyne. In this study, we put special emphasis on the determination of the impact of the fluorine content and the type of CF functionalities on the morphology, chemical and electronic structure, biocompatibility, electrical conductivity and possible applicability as anode materials for Li-ion batteries. The obtained results indicate that the character of C–F bonds influences the final properties of fluorinated materials. The polar C–F bonds are preferable for cell proliferation while CF_2_ groups are most suitable for battery devices, however, the appearance of PTFE-like units may have a negative impact on battery specific capacitance as well as on cell viability.

## Abstract 2

The appearance of PTFE-like nanochains in the structure of fluorinated N–graphyne strongly influences both electrochemical and biological properties.

## Graphical abstract

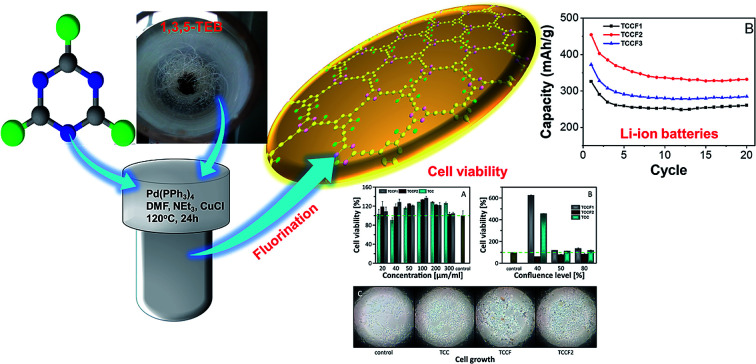

## Introduction

Graphyne-like nanostructures (GYs) belong to new two-dimensional carbon allotropes composed of sp- and sp^2^-hybridised carbon atoms, reported by Baughman *et al.*^1^ The graphyne structure can be viewed as a modification of graphene's structure where one-third of the carbon–carbon bonds are replaced by acetylenic bridges. Due to the unique sp–sp^2^ hybridisation of carbon atoms, uniform pores, and highly π-conjugated structure, 2D graphyne flat sheets are of interest to many research groups.^2^ Thanks to the flexible synthesis procedure and large number of aromatic compounds which could potentially be used as building blocks of new nanomaterials, GYs are considered as versatile materials with strong application potential.^3^

Numerous papers have been published discussing theoretical properties of GY-based materials and their applications. However, reported papers concerning synthesis and application of graphyne structures are handful and remains elusive practically.^4–6^

Introduction of heteroatoms is an efficient and essential method to adjust band gaps and electronic structures of carbon materials.^7–9^ Even though GYs themselves are interesting and promising materials, the presence of triple carbon–carbon bonds in their structure gives an opportunity for additional modifications through doping or functionalisation. These modifications results in deep changes in the structure and physicochemical properties of such modified materials.

Among numerous possible modification methods, fluorination, due to exceptional reactivity and electronegativity of fluorine, appears to be one of the most impressive and effective procedure allowing to receive completely new materials in relation to their precursors. Until now, fluorination process has been realised through various chemical routes with different fluorinating agents and resulted in distinct changes of electronic, optic and conductive nature of fluorinated materials.^10–17^

Fluorination has been widely used for various carbonaceous materials starting from amorphous activated carbons,^10^ through carbon nanotubes^11,12^ and fullerenes,^13^ ending on graphite^14^ and most recognisable graphene^15^ and graphene oxide.^16,17^ However, to our best knowledge, there are few reports focusing on direct fluorination of GYs. The recent papers deal, *e.g.* with graphdiyne-like structure made from trifluorobenzene precursor^18^ and graphdiyne-like structures fluorinated using XeF_2_ focused on their photoluminescence properties.^19^

In the present work, a new type of N–graphyne material built on 1,3,5-triethynylbenzene and triazine units was synthesised and subjected to fluorination reaction carried out with XeF_2_. Xenon difluoride was chosen as fluorination agent due to its high reactivity and selectivity towards organic compounds bearing double or triple bonds.^20^ Reaction of progressive fluorination of triple bonds, understood as transformation of acetylene units into double bounded CF=CF and single bonded, PTFE-like, CF_2_–CF_2_ units, was proceeded at room temperature. Reaction progress was controlled by varying concentration of XeF_2_. The impact of fluorine concentration as well as nature of C–F bonds on morphology, chemical and electronic structure, biological activity, conductivity and their applicability as Li-ion battery anodes was in-depth examined and the results of investigations are presented in the following sections in detail.

## Results and discussion

**Scheme 1:**
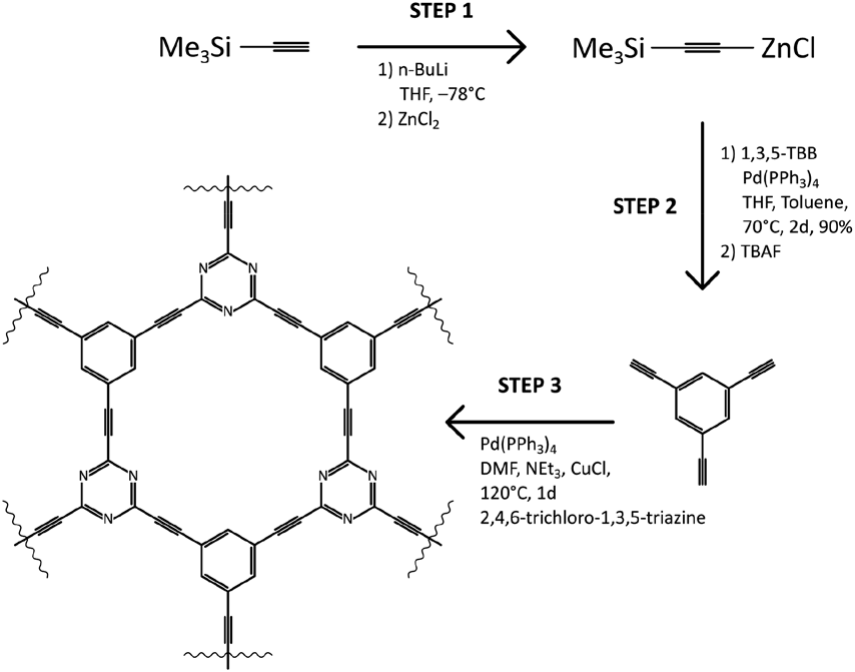
Detailed synthetic route towards TCC (top) and depiction of product formation in Step 3 (bottom).

The N–graphyne, called for our purpose TCC (triethynylbenzene coupling with cyanuric chloride), was synthesised upon solvothermal route in an equimolar cross-coupling reaction of 1,3,5-triethynylbenzene (1,3,5-TEB) and cyanuric chloride, depicted in Scheme 1. Obtained in Step 1 triethynylbenzene was recrystallized from melt carried out in tubular furnace at 400 °C and under nitrogen atmosphere. Obtained white needle crystals were investigated by single-crystal XRD to evaluate the structure of obtained 1,3,5-TEB and results are collected in ESI file (Fig. S1, S2 and Table S1†). Redetermined crystal data were deposited in CSD with CCDC number: 2025547 and are of better quality compared to the previously reported.^21^ That prepared substrate was subjected to Step 3 which resulted in a black jelly product with a thin layer of a clear solution on top of it, which was continuously washed by 5% HCl solution, Milli-Q water, ethanol, and acetone until the black product received. Further, TCC samples were fluorinated using XeF_2_ under ambient conditions, in a weight ratio of 1.5, 6.7 and 10 resulting in three fluorinated TCCs with increasing fluorine content: TCCF1, TCCF2 and TCCF3 respectively.

### Morphology

**Fig. 1:**
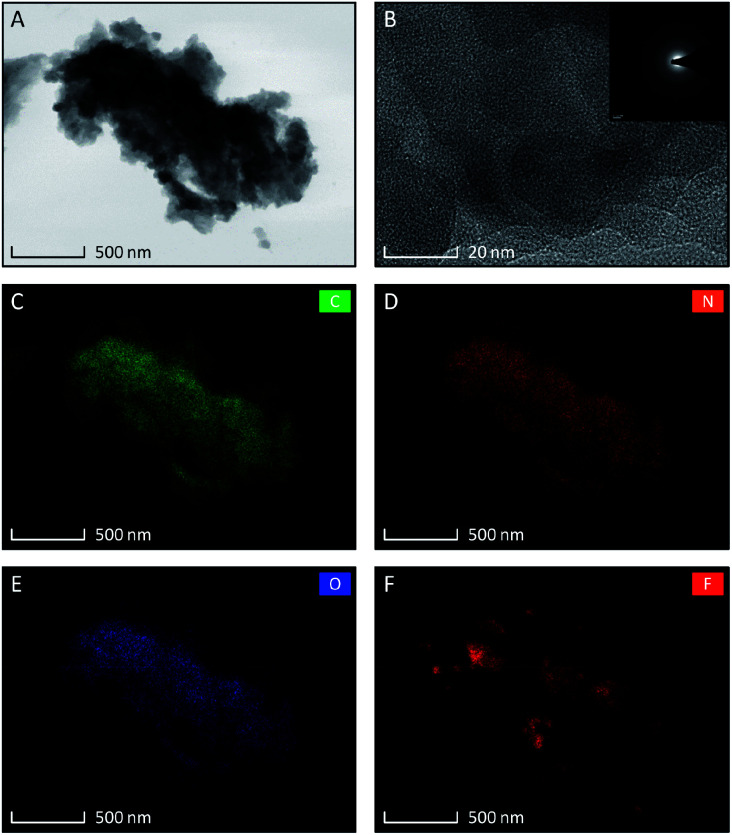
Set of TEM micrographs of fluorinated N–graphynes TCCF2 samples, (A) at low magnification; (B) at high magnification with inset showing SAED pattern. Low magnification images of EDX elemental distribution showing content of: carbon (C), nitrogen (D), oxygen (E), and fluorine (F).

**Fig. 2:**
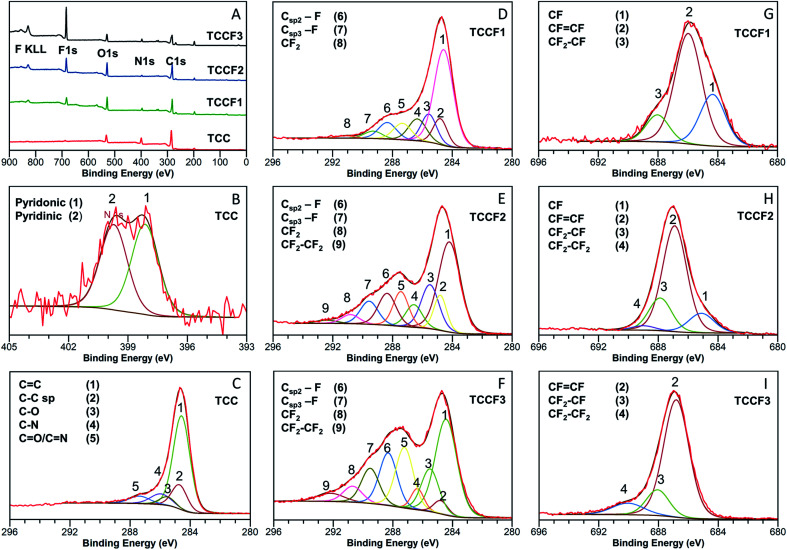
Survey XPS spectra of: (A) pristine and fluorinated N–graphynes. Magnification and deconvolution various bands of XPS spectra: C 1s (B) and N 1s (C) of pristine TCC; C 1s of TCCF1 (D), TCCF2 (E), TCCF3 (F); and F 1s of: TCCF1 (G), TCCF2 (H), TCCF3 (I).

The set of TEM images taken for TCCF2 sample are shown in Fig. 1A–F. The low magnification image (Fig. 1A) revealed that TCCF2 sample forms a cluster of micrometric irregular fragments with average dimensions of 1–15 μm. Closer observations showed that they are built from randomly arranged stacks of thinner layers. Furthermore, TCCs show porous and rather disordered structure confirmed by the SAED pattern presented in an inset of Fig. 2B. The rough chemical composition of the samples as well as the surface mapping was evaluated by EDX technique shown in Fig. 2C–F. Obtained elemental maps showed mostly homogeneous distribution of fluorine atoms (especially for TCCF1, Fig. S4†) on the whole surface, however, some agglomerations of fluorine atoms, presumably around inorganic impurities, can be seen for samples prepared using higher excess of XeF_2_. TEM observations also revealed the presence of oxygen and chlorine on the surface of the samples (Fig. 1E and F). The presence of chlorine could result from not fully reacted cyanuric chloride or residual chlorine from the catalyst used during the synthesis of TCCs (Table 1).

**Table 1:** Elemental content of pristine and fluorinated N–graphynes

Composition	TCC (at%)	TCCF1 (at%)	TCCF2 (at%)	TCCF3 (at%)
C	74.8	67.6	69.4	59.2
N	9.2	5.0	2.2	4.6
O	12.6	15.6	14.9	9.5
F	—	6.4	10.2	19.5
Cl	2.7	5.3	3.3	7.2

### Structural properties

The effects of fluorination on TCC can be seen already at first glance, as the reaction leads to a change in the samples' colour with increasing fluorine content from dark brown/black (TCC) through deep brown (TCCF1), pale brown (TCCF2) to yellow (TCCF3) (Fig. S3†). The structural changes as well as presence of new CF functionalities caused by fluorination can be observed using XPS, FTIR and solid state ^13^C and ^19^F NMR techniques.

Detailed investigation of elemental composition and functionalities of pristine TCC and its fluorinated derivatives was performed by XPS (Fig. 2A). Four core level peaks C 1s (285 eV), N 1s (399 eV), O 1s (530 eV) and Cl 2p (200 eV) are common for all TCCs and TCCFs, whereas fluorine built in the structure of the latter was confirmed by sharp F 1s peak around 686 eV and Auger F KLL peak at 830 eV. The elemental composition was determined by integration of above-mentioned core level peaks. It shows that successful fluorination was achieved to receive samples of increasing fluorine concentration of 6.38%, 10.16% and 19.48% for TCCF1, TCCF2 and TCCF3, respectively.

Fig. 2B shows N 1s XPS spectrum of TCC sample having one broad band which can be convoluted to two overlapping peaks at 398.28 eV and 400.15 eV characteristic for pyridinic and pyridonic nitrogen, whereas the latter peak could be a result of a partial hydrolysation or degradation of triazine rings.^22^

In contrary to rather uniform C 1s band found for TCC (Fig. 2C), the C 1s bands recorded for fluorinated samples show separated (TCCF2, TCCF3) or partly separated (TCCF1) broad peaks. First of these peaks lying in a range of 284–287.6 eV is common for TCCFs, pristine TCC and other known carbonaceous materials presenting mixed hybridisation and armed in various functional groups.^23,24^ Deconvolution of C 1s bands revealed that they are composed of several peaks found at 284.3 ± 0.2 eV, 284.9 ± 0.1 eV, 286.1 ± 0.2 eV and 287.1 ± 0.5 eV assignable to C=C, C≡C, C–N/C–O and C=O/C=N components, respectively. It turns out that, the intensity of bands at 284.9 eV has decreased from 33% (TCC) to 2.9% (TCCF3) as fluorine content increased indicating that fluorination took place predominantly on triple bonds of N–graphynes. The second broad band, so called CF region, appearing in a range of 288–292 eV can be attributed to carbon atoms directly linked to fluorine atoms, especially since their increasing intensity (Fig. 2D–F) follow the trend of increasing fluorine content in the sequence of TCCF1, TCCF2 and TCCF3. Again, deconvolution of C 1s bands helped to distinguish several peaks included in the CF region and appearing at *ca.* 288.3 ± 0.2 eV, 289 ± 0.3 eV, 291.3 ± 0.5 eV. The band near to 288.3 ± 0.2 eV is typically assigned to fluorine covalently bonded to sp^2^ carbon atoms. It could be noticed that the intensity of these units as well as CF functional groups bearing fluorine atoms attached to sp^3^ carbon units (289.5 eV) and the covalent CF_2_ functional groups appeared at *ca.* 291.1 ± 0.5 eV has been increasing progressively with increasing fluorine concentration. Finally, the small peaks seen at 292.4 ± 0.1 eV can be ascribed to CF_2_–CF_2_ units.^25^

Deconvolution of F 1s XPS spectra showed that they are built typically from three or four additional lines corresponding to different CF bonds present in the fluorinated samples. Isolated, *e.g.* surrounded by bare C atoms, semi-ionic C–F bonds (1) are seen in a range 684.6–685.2 eV, semi-ionic –CF=CF– units (2) present in range 686.5–686.9 eV, covalent –CF_2_–CHF– or –CF_2_–CH_2_– units (3) can be found in a range 688.1–688.8 eV; while the lines at ∼689.2 eV are assignable to PTFE-like covalent –CF_2_–CF_2_– units.^25^ The evolution of the nature of CF bonds with increasing fluorine content is clearly visible in the reduction of group (1) with a simultaneous increase in the share of groups (3) and (4). It should, however, be noted that bonds (2) remain the main core of C–F bonds occurring in fluorinated N–graphynes, regardless of the fluorine content. The distribution and amount of particular functional groups occurring on the TCC and TCCFs surface are collected in Tables S2 and S3† included in EIS file.

**Fig. 3:**
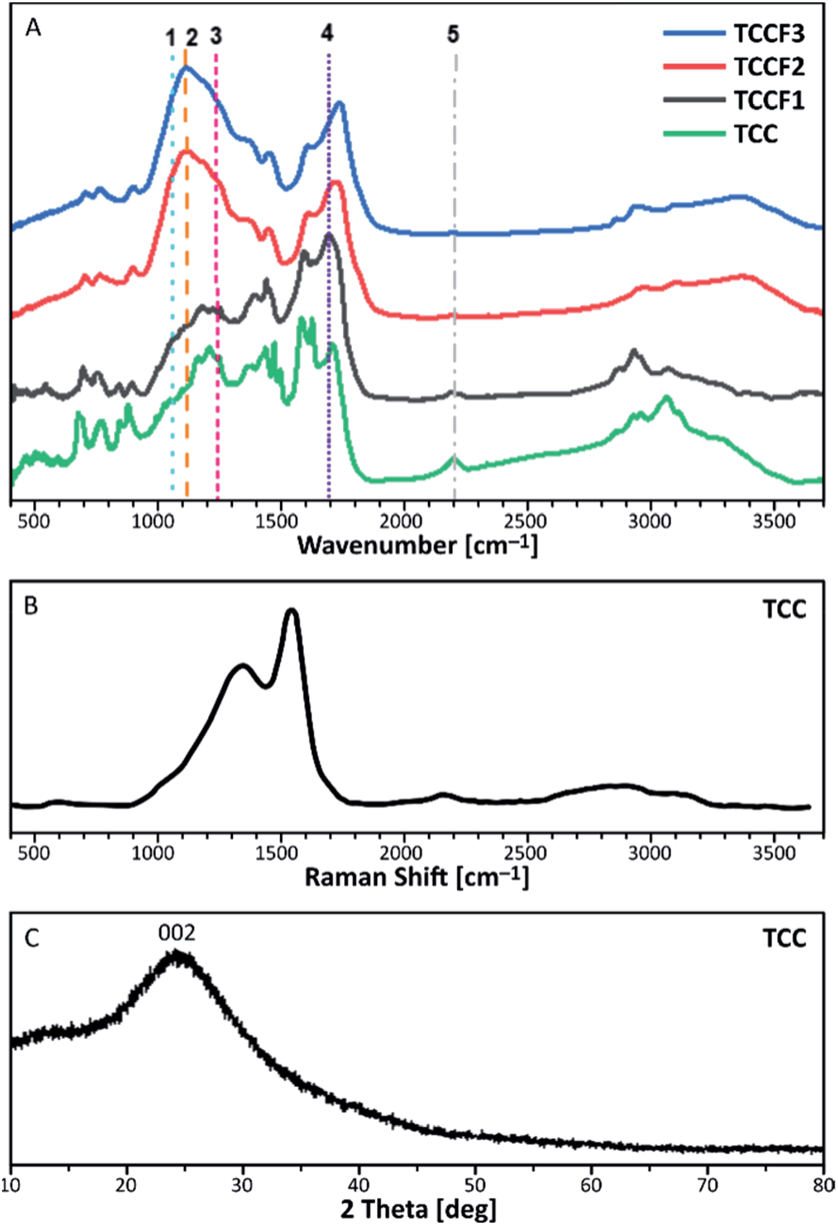
FTIR spectra of pristine and fluorinated N–graphynes (A); Raman Spectra (B) and XRD pattern (C) measured for of pristine N–graphyne (TCC).

FTIR spectra of pristine TCC and TCCFs are shown in Fig. 3A. Taking into account FTIR spectra measured and reported for fluorinated materials, the most significant and characteristic bands are those appearing in a range 1070–1250 cm^−1^.^15,26^ These bands can also be observed for fluorinated N–graphynes: TCCF1, TCCF2 and TCCF3. Increasing absorbance observed along with increasing fluorine concentration allow us to believe that these bands are predominantly correlated with different types of CF bonds present in the structure of TCCFs. In the case of TCCF1, wide shoulders at 1070 cm^−1^ and 1120 cm^−1^ and broad band 1170–1230 cm^−1^ (line 3) were distinguished. Adapting the concepts made for other fluorinated carbonaceous materials [ref. 27 and wherein], we could assign the band at 1070 cm^−1^ (line 1) to bulk CF groups surrounded by two or three bare C atoms, while the band at 1100 cm^−1^ (line 2) to two neighbouring CF groups surrounded by two bare C atoms. Moreover, the reaction of atomic fluorine with carbons of acetylenic units was confirmed by presence of vinylene groups –CF=CH– or –CF=CF– at 1690 cm^−1^ (line 4) as well as by significant decrease of absorbance at 2150 cm^−1^ (line 5) characteristic for –C≡C– bonds. The effect of fluorination became much more visible when high concentrations of XeF_2_ were applied as they resulted in reduction of a large number of isolated CF groups, which can be observed as decrease of the intensity of shoulders at 1070 cm^−1^ followed by a creation of chain-like fluorination pattern represented by maximum absorbance at 1120 cm^−1^. Furthermore, partial transformation of –CF=C(F)– to –CF_2_–CF_2_– units was confirmed by strong reduction of absorbance at 1690 cm^−1^. It should be mentioned that the lines occurring in the range 1170–1250 cm^−1^ should be treated with caution, as they may be derived from symmetrical and asymmetrical vibrations of CF_2_ groups, respectively, but also from the oxygen containing functional groups, overlapping those covalent C–F bonds.

Because of complete optical transparency of TCCFs to the green light (514 nm), it was impossible to receive significantly intensive Raman bands (Fig. S5†) for those samples as the energies of the lasers used were lower than the band gap of investigated samples.^28^ On the other hand, the Raman spectrum of pristine TCC is shown in Fig. 3B. It consists of three bands characteristic for carbonaceous materials. The shoulder at 1380 cm^−1^ is known as D band originating from a breathing mode of *k*-point photons of A_1g_ symmetry. The most significant band at 1580 cm^−1^, called G band, is formed due to disorder scattering of E_2g_ phonons by sp^2^ carbon atoms; the broad 2D band found in a range 2600–2900 cm^−1^ is treated as an overtone of D band and exhibits a strong frequency dependence on the excitation laser energy. The last small band at 2145 cm^−1^ is a common band for graphyne and graphdiyne-like structures coming from –C≡C– units. Deconvolution of D and G bands helped to evaluate the areas ratio known as *I*_D_/*I*_G_, which brings an information about the ordering rate of carbonaceous nanostructures.^29^ In the case of N–graphyne, the *I*_D_/*I*_G_ ratio was equal to 2.86 suggesting mostly disordered structure, later confirmed by powder XRD shown in Fig. 3C. The XRD pattern contains one broad and noisy signal at 2*θ* = 24.44°, referring to 002 reflection in graphite structure.^30^ The broadness of the signal as well as the absence of *h*0*l* and *hkl* reflections in the pattern of N–graphyne indicates the lack of graphitic ordering and rather amorphous nature of N–graphynes.

**Fig. 4:**
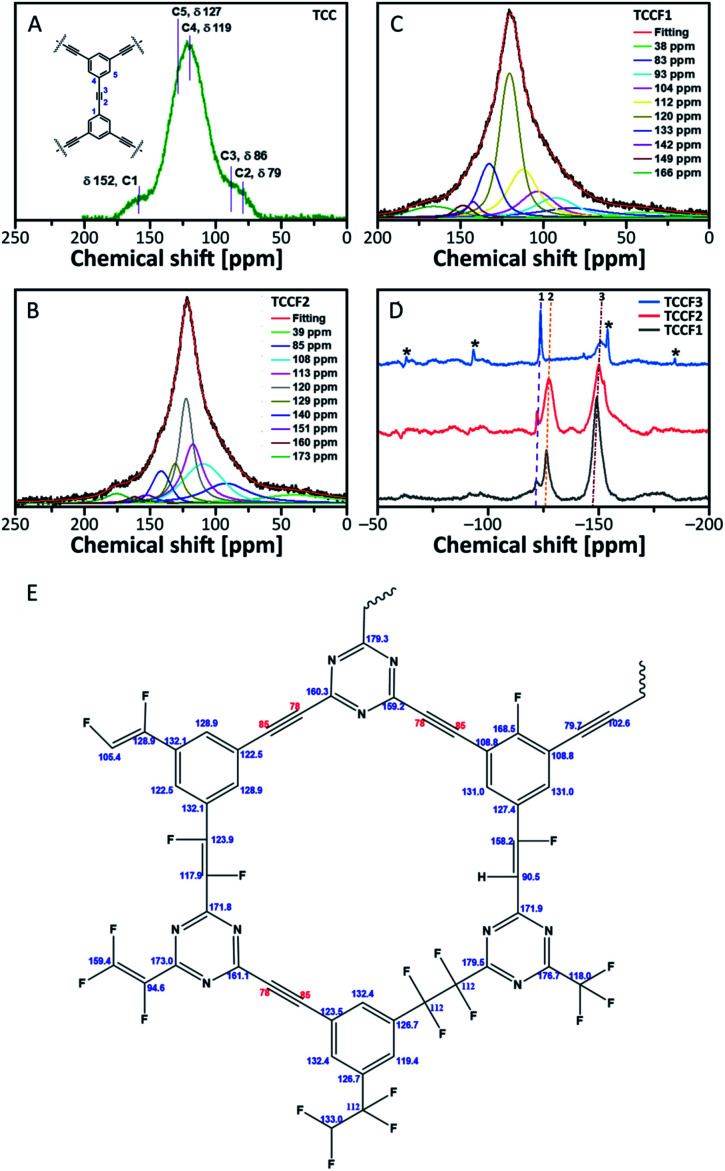
^13^C solid state NMR of pristine TCC (A), TCCF1 (B) and TCCF2 (C); ^19^F solid state NMR of fluorinated N–graphynes, wherein 1 is CF_2_, 2 is CF=CH, 3 is CF=CF and asterisks are rotational sidebands of 3 (D); the map of CF bonds made up during fluorination (E).

^13^C NMR spectra of fluorinated N–graphynes are presented in Fig. 4B and C. It could be noticed that all recorded spectra do not differ strongly from each other. What is more, they were very similar to the spectrum of pristine TCC shown on Fig. 4A, where the inset picture presents a fragment of the investigated molecular structure with numbered bonds (C1–C5) visible at NMR spectrum: 152 ppm (C1), 76–90 ppm (C2 and C3) and 118–127 ppm (C4 and C5). The spectra of fluorinated samples comprised broad asymmetric peaks with several shoulders. Due to broad and jagged spectra presenting numerous overlapping lines causing difficulties and uncertainties upon interpretation, Voight line shape deconvolution^31^ have been conducted using OriginPro 2020 software in order to determine the chemical structure of the tested materials. Obtained results revealed that spectra are built from several signals of different chemical shifts representing different types of C–C and C–F bonding. The carbons included in acetylenic units –**C**≡**C**– give a chemical shift in a range of 78–91 ppm (discussed C atoms are bold); this line however can overlap with C sp^3^ carbons directly bonded to fluorine recorded at chemical shift of 86 ppm. The chemical shifts in a range of 34–40 ppm can be assigned to carbons in –**C**H_2_–CF_2_– units. The main peak at 118–120 ppm was assigned to aromatic carbons with hybridisation sp^2^, for our purposes further named as C_ar_. These bands, however, comprise several shoulders at chemical shifts of 104–108 ppm (C_ar_–**C**H=CF–C_ar_) and 112–113 ppm (C_ar_–**C**F_2_–**C**F_2_–C_ar_). Signals at 133 ppm can be assigned to C_ar_ linked directly to –CF=CF– unit, whereas those at 140 ppm to C_ar_ bonded to –CF_2_–CF_2_– linkers. The peaks with chemical shift at 169 ppm belong to C_ar_ of triazine ring linked to one of aforementioned CF units. Deconvoluted ^13^C NMR spectra for TCCF3 is depicted in ESI file (Fig. S6†). All possible fluorinated places have been depicted in Fig. 4E.

^13^C NMR spectra prepared for TCCFs show significant differences between these materials and *e.g.* fluorographene. For the latter, usually, two distinct chemical shifts can be distinguished, first at 82–86 ppm characteristic for C sp^3^–F groups and the second one at 132 ppm assignable to the interactions between sp^2^ carbon and fluorine atom from the neighbouring CF groups.^32^

The formation of carbon–fluorine bonds after fluorination conducted with various concentration of XeF_2_ was confirmed by solid state ^19^F NMR and the normalised spectra are shown in Fig. 4d. For all the samples, three main signals at chemical shifts of −122 ppm (CF_2_), −127 ppm (–CF=CH–) and −150 ppm (–CF=CF–) are observed with the intensity of each type of a signal clearly depended on fluorine content. In the case of TCCF1, the bands at −150 ppm are dominant whereas the increasing fluorine concentration leads to a decrease of its intensity with simultaneous strengthening intensities of signals at −122 ppm noticed for samples TCCF2 and TCCF3.

Based on this finding, it can be concluded that CF=CF systems are created in the first place, while higher amounts of available fluorine can further fluorinate them to CF_2_–CF_2_ systems. The signals at chemical shift of −127 ppm, found for TCCF1 and TCCF2, completely disappeared in the case TCCF3 likely due to the transformation of –CF=CH– bonds to CF_2_–CHF and/or CF_2_–CF_2_ units. Obtained results well support the findings of XPS and FTIR.

### Electronic structure

**Fig. 5:**
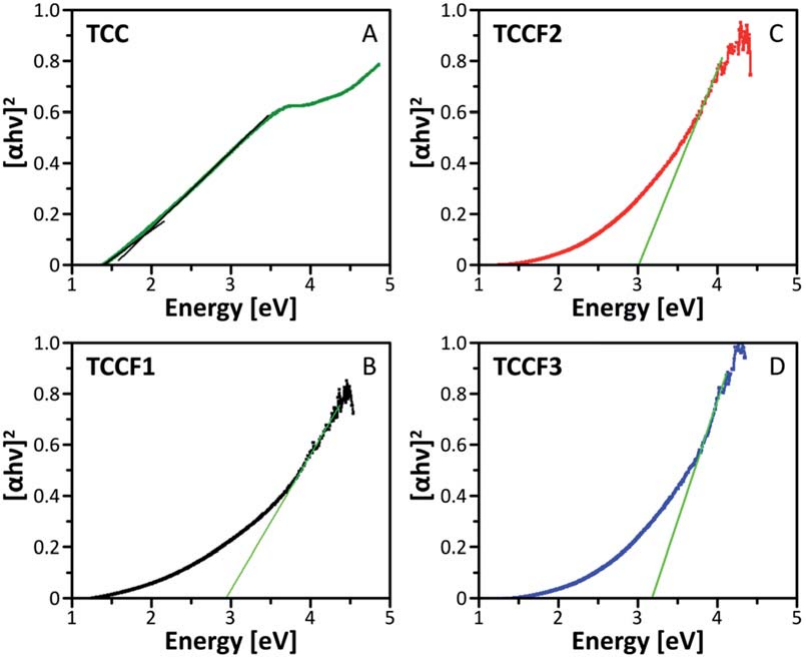
Tauc plots prepared for pristine (TCC) and fluorinated N–graphynes (TCCF1, TCCF2 and TCCF3).

The Tauc plots shown in Fig. 5 were prepared in order to determine the optical gap of investigated materials. The exponent *r* in (*αhv*)1/*r* formula fixed at ½ turned out to be the most suitable for all the investigated samples suggesting allowed direct transitions occurring in all these materials regardless the fluorination rate. The optical gap of pristine TCC is estimated to be 1.86 eV what nicely corresponds with the values of optical gap found for other reported nanostructures containing conjugated triazine and benzene rings^33^ as well as triazine–graphdiyne nanostructures.^34^ As expected, fluorination of TCC leads to significant widening of the optical gap, resulting in the values of 2.93 eV, 3.03 eV and 3.17 eV observed for TCCF1, TCCF2 and TCCF3, respectively. The reason of such feature lies in increasingly stronger covalent nature of CF bonds associated with the augmentation of fluorine concentration as well as the occurrence of electron–hole and electron–electron interactions resulting from the impact of UV light on the fluorinated samples.

HOMO–LUMO (shortly HL) gaps of fluorinated TCCs, were determined computationally using DFT method using Gaussian 16^35^ by hybrid functional B3LYP^36^ with Def2SVP basis set.^37^ The HL gap has been determined from the eigenvalues of HOMO and LUMO Kohn–Sham orbitals. The structure considered in these experiments are shown in Scheme 1S.† Briefly, a model structure of TCC consisting of 10 benzene rings and 3 triazine rings connected by single acetylene linkers was employed and the whole structure formed a monolayer of combined three macrocycles. That prepared model structure served as a starting point for new structures with attached fluorine atoms in the number from 2 to 48 arranged in different bond configurations (–CF=CF– and –CF_2_–CF_2_–). Additionally, the calculations of HL band gap of a structure with erased triazine rings and containing 12 fluorine atoms were also conducted. All considered structures with a full set of results are presented in ESI file.† The HL gap calculated for the model structure was equal to 3.43 eV, whereas the HL gaps calculated for fluorinated derivatives lied in a range of 3.12–3.34 eV and were related to fluorine concentration, CF bond nature as well as aromatic nature of fluorinated sample. Our findings meet well with band-gap energy (3.13 eV) found for recently reported 2D halogen-substituted graphdiyne.^38^ Although calculations revealed that band gap can be modulated by the fluorine concentration and CF bond nature, the change in a band gap is rather moderate and does not exceed 0.4 eV. It turns out that the most significant change in the band gap (0.8 eV) was noticed when triazine ring was erased from N–graphyne structure. In this case, HL gap increased to 3.78 eV making it very close to experimental values found for fluorographene^39^ or fluorinated graphene oxide.^16^

Unfortunately, discrepancy between theory and experiment could be seen in overall trends of gap modification. In contrary to the experimentally estimated optical gap, the theoretical considerations disclosed that HL gap decreased with decreasing fluorine concentration. The explanation of such behaviour may be attributed to defects and corrugations occurring in real samples. Furthermore, the DFT calculations did not consider photon-assisted electron–hole and electron–electron interactions. The agreement between theory and experiment could likely be improved in the future by applying other calculation model such as the GW–Bethe–Salpeter Equation (GW–BSE) allowing to apply rectification of the gap by comprising electron–electron and electron–hole interactions.^40^

**Fig. 6:**
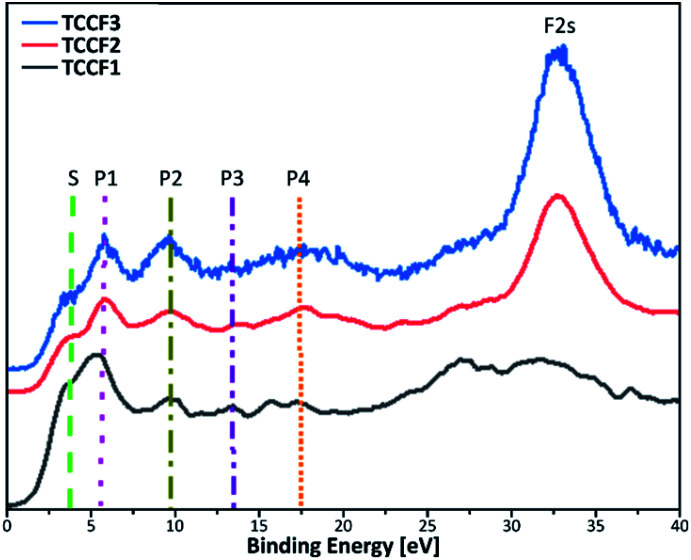
Valence band XPS spectra of fluorinated N–graphynes. Additional lines are drawn to visualise the bands described in the text.

Fig. 6 shows Valence Band XPS (VB XPS) spectra, measured for fluorinated N–graphynes, with six characteristic lines in a range 3.8–32 eV observed for all investigated samples.

Given the high convergence of the results obtained in this work with previously reported ones for various types of fluorinated materials,^27,41,42^ the π states of aromatic regions can be ascribed to a shoulder at *ca.* 3.8 eV (S), a band at 5.5 eV (P1) represents interactions of fluorine orbitals being nearly parallel to the sheets with TCC σ system, a band at 9.6 eV (P2) manifests non-bonding fluorine states, and a band at 13.6 eV (P3) is assigned to C–F bonding. The bands at 17.6 eV (P4) and 32 eV (F 2s) result from the C 2s and F 2s states, respectively. For all the samples, the peaks' positions are nearly the same, however, show moderate variations in intensity (Table 2). The valence band energy taken as offset of the VB XPS are in a range 1.60–1.87 eV showing a moderate shift to higher values with increasing fluorine concentration. The values of conductive band minimum energy estimated as a sum of valence band maximum energy and the appropriated optical band gap are collected in Table 2.

**Table 2:** Peak positions extracted from valence band XPS data

VB XPS	TCCF1 [eV]	TCCF2 [eV]	TCCF3 [eV]
Shoulder (S)	3.8	3.7	3.8
Peak 1 (P1)	5.5	5.5	5.7
Peak 2 (P2)	9.6	9.5	9.5
Peak 3 (P3)	13.5	13.7	13.8
Peak 4 (P4)	17.1	17.7	17.6
F 2s	32.2	32.5	32.0
VB	1.6	1.8	1.9
CB	4.5	4.8	5.1

### Electric properties

**Fig. 7:**
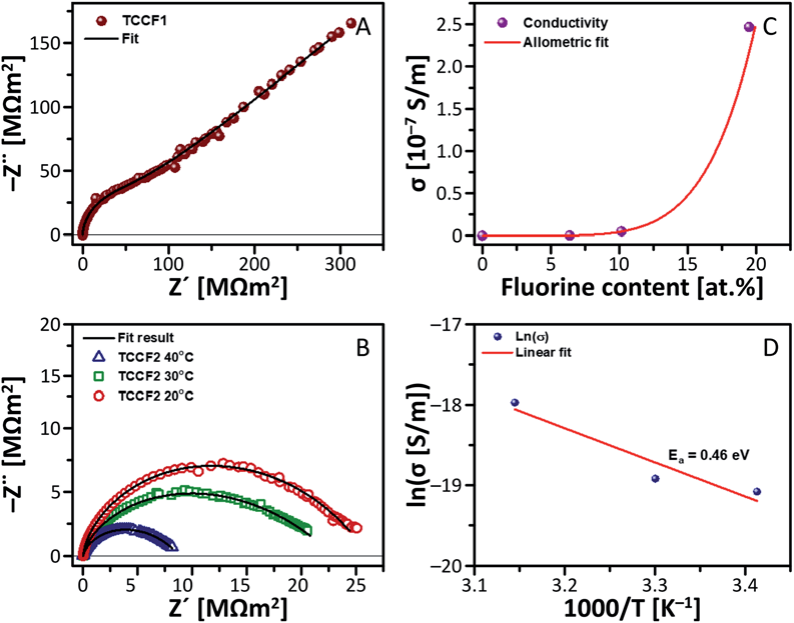
Electric properties of N–graphyne and its fluorinated derivatives: (A) Nyquist plot with Warburg impedance measured for TCCF1; (B) dependence of electrical conductivity on fluorine content of TCCFs; (C) Nyquist plot measured for TCCF2 in different temperatures; (D) Arrhenius plot derived for TCCF2.

Conductivity of the samples was investigated by electrochemical impedance spectroscopy (EIS) using IMPED CELL, an in-house designed and patented device made of hardened stainless steel, providing simultaneous control of pressure up to 2000 MPa, and temperature in the rage of 0–80 °C.^43,44^Fig. 7A and B shows an exemplary Nyquist plot registered for TCCF1 along with equivalent circuit used. Fig. 7b shows Nyquist plots of TCCF2 measured in three different temperature: 20 °C, 30 °C and 40 °C. For each sample, a single semicircle or deformed semicircle was observed. However, for some fluorinated samples semicircles were followed by a Warburg impedance (line tilted at 45°) at lower frequencies related to the charge accumulation at blocking electrodes, suggesting some ionic conductivity of these samples. The arc is identified as a bulk property from the fact that it passes through the Z′–Z′′ origin, and from the associated capacitance. The deformed semicircle may occur due to difference in time constants *e.g.* grain interior, grain boundaries conductivity and reactions with electrode material in the low frequency range as well. EIS measurements showed that both pristine and fluorinated TCCs exhibit very high resistivity in the range of MΩ m^2^ which translate into very low electrical conductivity. Fig. 7C presents relationship between electrical conductivity and fluorine concentration in the samples.

The electrical conductivity of pristine TCC was equal to 2.31 × 10^−9^ S m^−1^ and addition of 6.38 at% of fluorine yielded in enhancement of the electrical conductivity by almost 1.5 times. Further increase of fluorine concentration resulted in further enhancement of conductivity reaching the level higher by about 1–2 orders of magnitude than conductivity of TCC sample. Obtained results fitted with the low power function can be expressed by equation: *y* = *a* × *x*^*b*^, where scaling exponent, *b*, is 5.78 and initial growth index, *a*, is equal to 7.0 × 10^−8^.

Temperature resolved EIS measurements (293–318 K) showed a dependence of electrical conductivity on temperature. Fig. 7D shows temperature-dependent conductivity of TCCF2 exhibiting a linear behaviour in an Arrhenius plot, which enables determination of activation energy of conductivity of TCCF2 to be 0.46 eV. The energy activation level as well as a shape of semicircles indicate that electric transport, in this range of temperature, appears to be realised on electronic route. The enhancement of electrical properties of TCCFs seems to be directly caused by increasing fluorine content, linked with carbon mostly *via* semi-ionic –CF=CF– units. In such case, fluorine being an electron acceptor acts as a p-dopant for investigated materials and the electrical conductivity is realised on electron–hole transport way between carbon and fluorine. Furthermore, the increase of temperature results in higher mobility of polar molecules, in consequence leading to an increase of the dielectric constant shown in Fig. S8.†

### Electrochemistry

**Fig. 8:**
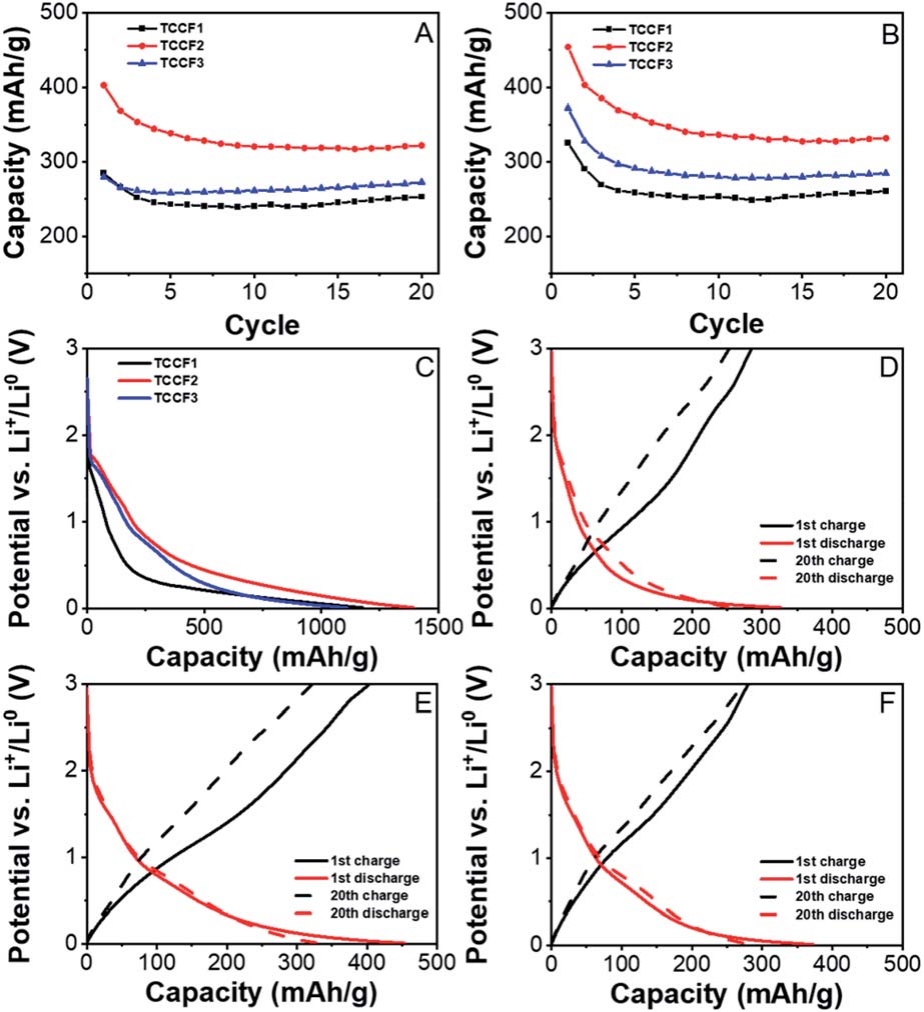
Cyclability of TCCFs during charge (A) and discharge (B) processes and charge/discharge curves for TCCF powders: initial discharge (C), 1st (solid line) and 20th (dashed line) cycle charge/discharge curves of TCCF1 (D), TCCF2 (E) and TCCF3 (F).

Fig. 8A and B show the results of galvanostatic charge (A) and discharge (B) cycling for TCCFs. All compounds delivered high discharge capacity during preliminary discharge, reaching 1175.5 ± 38.3 mA h g^−1^, 1394.3 ± 42.8 mA h g^−1^ and 1109.5 ± 16.6 mA h g^−1^ for TCCF1, TCCF2 and TCCF3, respectively. After the initial discharge, the capacities dropped drastically to 285.0 ± 8.2/325.8 ± 8.5 mA h g^−1^, 402.7 ± 15.4/454.3 ± 27.5 mA h g^−1^ and 280.0 ± 34.6/372.4 ± 46.1 mA h g^−1^ during 1st charge/discharge cycle for TCCF1, TCCF2 and TCCF3, respectively. The irreversible capacity during preliminary discharge, as a result of Solid Electrolyte Interphase (SEI) layer formation on the surface of the electrodes, can be attributed to Li^+^ reaction with electrochemically active CF groups.^45^ In consequence, received capacities are significantly lower than those found for F-substituted graphdiyne or triazine–graphdiyne materials.^21,34,46^ After five additional cycles the electrochemical performance of the cells stabilised and after 20th cycle the powders delivered specific capacities of 253.1 ± 15.2/260.7 ± 16.2 mA h g^−1^, 322.2 ± 60.1/332.1 ± 62.4 mA h g^−1^ and 272.7 ± 34.0/284.8 ± 36.8 mA h g^−1^ for TCCF1, TCCF2 and TCCF3, respectively. From all the examined samples, TCCF_2_ showed the highest specific capacities. This might be the result of CF_2_ groups formation, leading to increased vacancies for Li^+^ storage.^47^ It turns out that type of CF_2_ bonding could influence the final electrochemical properties of fluorinated materials as we noticed that higher number of CF_2_–CF_2_ units in the structure of TCCF3 resulted in drop of its specific capacity. Cyclability of all the examined samples had similar values, within the margin of error, reaching 88.8 ± 3.6/80.0 ± 3.6%, 79.6 ± 12.4/72.5 ± 9.8% and 97.4 ± 3.6/76.5 ± 3.8% of capacity retained after 20 cycles, for TCCF1, TCCF2 and TCCF3, respectively.

Fig. 8C–F show charge/discharge profiles of TCCF samples. The charge/discharge profiles have similar courses as described previously in the literature.^45–48^ The discharge curves show a steady decline in electrode potential during reduction for every examined sample. One can see a short plateau emerging below 1.00 V (*vs.* Li^+^/Li^0^), which quickly fades into another decline up to *ca.* 0.50 V (*vs.* Li^+^/Li^0^). This process is a result of SEI formation and reactions with CF groups present in TCCs. Below 0.5 V (*vs.* Li^+^/Li^0^), insertion of Li^+^ ions into the fluorinated N–graphyne interlayers starts to take place. During charge processes, for every examined sample, there is almost steady increase of electrode potential in the entire range of 0.01–3.00 V (*vs.* Li^+^/Li^0^), with no distinct plateaus, which is consistent with previous reports on alkali ion storage in graphene-like structures.^45,47^

### Biocompatibility

**Fig. 9:**
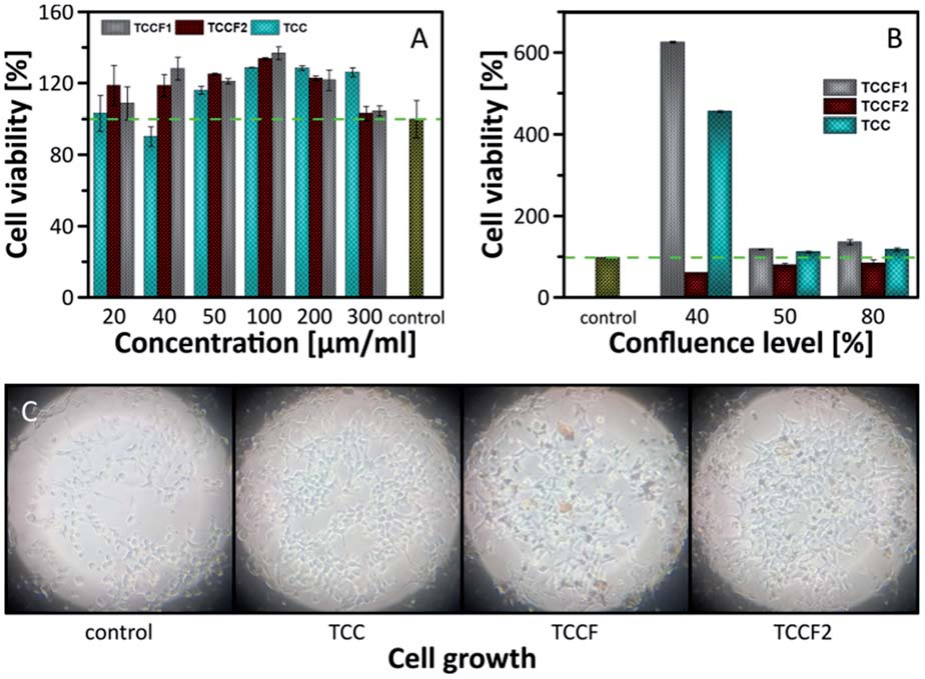
Cell viability investigated on: (A) confluent cells; (B) cells with 30%, 50% and 80% of confluence; (C) photos of growing cells taken for 30% of confluence.

Biological tests, cell viability and antibacterial activity, were conducted in order to determine the biocompatibility of pristine TCC and TCCFs. Using *Escherichia coli* strain, no antibacterial properties of the materials have been found (Fig. S10†), however, it seems that cell viability is significantly affected during 24 h treatment. Fig. 9 presents the results of human embryonic kidney (HEK) cell viability tests. The obtained results prove that all investigated materials did not show cell toxicity within considered concentration range. On the contrary, the treated cells exhibited higher number of living cells in a range 103–133% for the most of the samples compared to the data from control untreated HEK cell culture. It turns out that sample concentration had some effect on cell viability, *e.g.* the cell viability has been progressively increasing reaching maximum for cell cultures treated with 100 μg ml^−1^ of investigated materials, whereas further increasing material concentration yielded in the drop of living cells.

The results discussed above were received in an experiment conducted on confluent cell cultures, thus, we could speculate that treated cells continue the process of proliferation or does not undergo the process leading to decrease of living cells as in control cells.

In order to investigate the impact of TCCF materials on cell proliferation, the cells cultures with 30%, 50% and 80% of cell confluences were employed. The results presented in Fig. 9A show the diverse influence of the tested materials on cell proliferation. Considering cell cultures with 50% and 80% of confluence, the number of living cells was on the same level as those presented in Fig. 9A, what suggests that samples may assist in the proliferation, but only to a limited extent. The most significant impact of investigated materials on cell growth was observed for cell cultures at 30% confluence level seen as six-fold (TCCF1) and four-fold (TCC) increase in cell densities. The biological importance of fluorinated carbonaceous materials lies in highly polar C–F bonds being expected to induce biological responses. The introduction of CF groups reduces surface energy of the modified materials leading to a change in wetting features which results in better cell adhesion and proliferation.^49^ The reason of low cell proliferation grown on TCCF2 may be related to decrease of overall number of CF bonds and appearance of –CF_2_–CF– and –CF_2_–CF_2_– units on the surface.

The CF_2_ bonds are characterised by a smaller dipole moment than C–F bonds, thus, smaller value of the total electric field acting on the surface. Weak electrostatic interactions and a lower polarity of CF_2_ groups lead to poorer cell adhesion and, consequently, much weaker cell proliferation comparing to highly polar CF bonds. In the case of pristine TCC samples the high cell proliferation may result from electrostatic interactions between oxygenated functional groups of TCC and fetal bovine serum used in a cell cultures as proteins contained in the serum directly affect cell adhesion and their morphology.^49,50^Fig. 9C shows micrographs of cell cultures treated with investigated materials. In comparison to round shaped control cells, those grown on fluorinated samples are elongated. The latter feature is directly related to electrostatic interactions occurring at the interface of cell and fluorinated N–graphynes.^50^

## Experimental

### TCC synthesis

N–graphyne was produced in 3-step reaction shown in Scheme 1. In first step ZnCl@trimethylsilylacetylene complex was synthesised and coupled with tribromobenzene in presence of Pd(PPh_3_)_4_ as a catalyst. Obtained tris[(trimethylsilyl)ethynyl]benzene was deprotected using 1 M TBAF, vacuum dried and recrystallised in tubular furnace at 400 °C under nitrogen atmosphere. Received white needle-shaped crystals of 1,3,5-TEB were finally used in cross-coupling reaction with cyanuric chloride. The received product was washed with 5% HCl, Milli-Q water, ethanol, and acetone.

### TCC fluorination

Three batches of solid TCC equal in mass (150 mg) were fluorinated by adding of XeF_2_ onto TCC/CH_2_Cl_2_ (dichloromethane) frozen mixture in a weight ratio varying from 1.5 up to 10. Reaction mixtures were slowly warmed to ambient temperature resulting in three fluorinated TCCs with increasing fluorine content: TCCF1 (ratio 1.5), TCCF_2_ (ratio 6.7), and TCCF3 (ratio 10). Direct reaction between the TCC and XeF_2_ without the presence of CH_2_Cl_2_ should be avoided!! such attempt resulted in the violent explosion inside the dry-box. Detailed description of these experiments is available in ESI file.†

### Physicochemical characterisation

The crystals of 1,3,5-TEB were investigated by single crystal XRD at 130(2) K on a Bruker D8 Venture Photon II diffractometer equipped with a TRIUMPH monochromator and a MoK_α_ fine focus sealed tube (*λ* = 0.71073 Å). A total of 1608 frames were collected with Bruker APEX3 program [S1]. The frames were integrated with the Bruker SAINT software package [S2] using a narrow-frame algorithm. Morphology of pristine and fluorinated N–graphyne samples were investigated by transmission electron microscopy (TEM), energy-dispersive X-ray spectroscopy (EDX) and selected area electron diffraction (SAED) using Thermo Scientific TALOS F200X microscope, working with electron excitation energy of 200 keV. Prior the tests, powdered samples were ultrasonically dispersed in acetone and transferred onto a 200-mesh carbon-coated copper grid (Electron Microscopy Science, USA). Elemental composition and functionalities of the discussed materials were investigated by X-ray photoelectron spectroscopy (XPS) recorded on Kratos Axis Supra spectrometer, equipped with a monochromatic Al Kα radiation source (1486.7 eV) operating at 150 W. The instrument work function was calibrated to give a BE of 84.0 ± 0.1 eV for the 4f_7/2_ line of metallic gold and the spectrometer dispersion was adjusted to give a BE of 932.62 eV for the Cu 2p_3/2_ line of metallic copper. Fourier transform infrared spectroscopy (FT-IR) characterisation was performed with Bruker Vertex 80 spectrophotometer using a KBr pellets. Solid-state ^13^C and ^19^F NMR spectra were collected with Agilent 700 MHz spectrometer at room temperature using 3.2 mm HXY triple-channel probe under MAS rate of 20 kHz. Raman spectra were measured on a Horiba T64000 spectrometer using a 20× objective lens and a laser excitation at 514.5 nm. XRD patterns were recorded by Panalytical X'Pert Pro diffractometer with a 2*θ* range of 5–90° with 0.1° step. UV-vis absorption spectra of ethanol dispersions of investigated materials were recorded on a Shimadzu UV-1800 spectrophotometer at room temperature. Electrical impedance spectroscopy (EIS) measurements were carried out using a Solartron 1260A Frequency Response Analyser equipped with 1296A dielectric interface and coupled with IMPED CELL sample holder. EIS spectra were collected within the frequency range of 10^7^ to 10^−2^ Hz and AC amplitude of 10–1000 mV.

### Electrode preparation

TCCF samples were firstly ground with Vulcan XC72R (Cabot) conductive carbon in an agate mortar for 20 minutes. Then, the solid mixture was transferred to a conical flask, 5 wt% polyvinilidene fluoride (PVDF, Alfa Aesar) solution in *N*-methylpyrrolidone (Sigma-Aldrich) was added dropwise and the received suspension was stirred on a magnetic stirrer for the next 4 hours. So prepared slurry was then cast onto a copper foil by a doctor blade technique, preliminarily dried at 55 °C in air, and vacuum dried at 120 °C overnight. Round, 9 mm in diameter, electrodes where then cut, pressed on the hydraulic press at 6 *t* for 1 minute, weighted, dried under vacuum at 120 °C overnight and transferred to argon filled glove-box (MBraun) for the cell assembly. The obtained electrode composition was 8 : 1 : 1 (wt.) TCCF : Vulcan : PVDF.

### Electrochemical measurements

Prepared electrodes were assembled into three-electrode Swagelok® system, with working electrode made of TCCF, both counter and reference electrodes made of metallic lithium (Sigma-Aldrich) and Celgard® 2325 separator soaked in electrolyte solution of 1 M LiPF_6_ in ethylene carbonate/dimethyl carbonate (1 : 1, v/v). Galvanostatic charge/discharge cycling was performed on multichannel battery tester ATLAS 1741 (Sollich) between 0.01 and 3.00 V (*vs.* Li^+^/Li^0^) at room temperature. The cells were preliminarily discharged at current rate of 400 mA g^−1^ and then charged/discharged for 20 consecutive cycles at the same current rate.

### Biological test

The cell viability was performed on HEK293 cells (human embryonic kidney) using the CellTiter Blue Reagent (CTB, Promega, Poland) and antibacterial activity tests were carried out on *Escherichia coli* microorganisms using agar disk-diffusion method.

### Computations

DFT calculations of electronic structure were conducted with the Gaussian 16 software and using the hybrid B3LYP functional calculations and Def2SVP basis set.

Further details regarding material's synthesis and applied measurements methodology is included in ESI file.†

## Conclusions

The present work focuses on synthesis, modification and investigation of new N–graphyne material prepared *via* solvothermal reaction of 1,3,5-triethynylbenzene with cyanuric chloride. The received N–graphyne was further modified by fluorination carried out at ambient conditions using xenon difluoride as fluorinating agent. Detailed spectral analysis showed the evolution of CF bonds nature when different amounts of XeF_2_ were used upon fluorination, starting from isolated CF groups through CF=CF or –CF=CH– units and CF_2_–CF, –CF_2_–CH_2_– ending on PTFE-like linkers. Despite the evolution of CF bonds, the semi-ionic –CF=CF– units remained dominant for all investigated fluorinated samples.

The diversity of CF bonds has a direct impact on physicochemical, electronic or electrical properties. The presence of semi-ion bonds translates directly into the electrical properties of the materials tested. Fluorinated N–graphyne can stimulate cells to grow. Interestingly, it seems that the type of CF_2_ groups have an influence on electrochemical properties. In our case, the increase in the share of CF_2_–CF_2_ groups have contributed to a decrease in the capacity of Li-ion batteries.

Concluding, results presented in this study proved that fluorination of N–graphynes is an effective way to receive versatile materials with desired properties potentially applicable in many field of science and industry.

## Conflicts of interest

There are no conflicts to declare.

## Supplementary Material

RA-010-D0RA08143D-s001

RA-010-D0RA08143D-s002

## References

[cit1] Baughman R., Eckhardt H., Kertesz M. (1987). J. Chem. Phys..

[cit2] Inagaki M., Kang F. Y. (2014). J. Mater. Chem. A.

[cit3] Xie C., Wang N., Li X., Xu G., Huang C. (2020). Chem.–Eur. J..

[cit4] Song Y., Li X., Yang Z., Wang J., Liu C., Xie C., Wang H., Huang C. (2019). Chem. Commun..

[cit5] Chen T., Li W. Q., Chen X. J., Guo Y. Z., Hu W. B., Hu W. J., Liu Y. A., Yang H., Wen K. (2020). Chem.–Eur. J..

[cit6] Yang C., Li Y., Chen Y., Li Q., Wu L., Cui X. (2019). Small.

[cit7] Muz I., Kurban M. (2020). J. Alloys Compd..

[cit8] Muz I., Kurban M. (2019). J. Alloys Compd..

[cit9] Kurban M. (2018). Optik.

[cit10] Wang X., Harris H. R., Bouldin K., Temkin H., Gangopadhyay S., Strathman M. D., West M. (2000). J. Appl. Phys..

[cit11] Adamska M., Narkiewicz U. (2017). J. Fluorine Chem..

[cit12] Zhang W., Bonnet P., Dubois M., Ewels C. P., Guerin K., Petit E., Mevellec J. Y., Vidal L., Ivanov D. A., Hamwi A. (2012). Chem. Mater..

[cit13] Szala-Bilnik J., Costa Gomes M. F., Padua A. A. H. (2016). J. Phys. Chem. C.

[cit14] Asanov I. P., Bulusheva L. G., Dubois M., Yudanov N. F., Alexeev A. V., Makarova T. L., Okotrub A. V. (2013). Carbon.

[cit15] Chronopoulos D. D., Bakandritsos A., Pykal M., Zboril R., Otyepka M. (2017). Appl. Mater. Today.

[cit16] Zhao F.-G., Zhao G., Liu X.-H., Ge C.-W., Wang J.-T., Li B.-L., Wang Q.-G., Li W.-S., Chen Q.-Y. (2014). J. Mater. Chem. A.

[cit17] Feng W., Long P., Feng Y., Li Y. (2016). Adv. Sci..

[cit18] Shen X., He J., Wang K., Li X., Wang X., Yang Z., Wang N., Zhang Y., Huang C. (2019). ChemSusChem.

[cit19] Xiao W., Kang H., Lin Y., Liang M., Li J., Huang F., Feng Q., Zheng Y., Huang Z. (2019). RSC Adv..

[cit20] Liu G. , Fluorination of Alkenes and Alkynes for Preparing Alkyl Fluorides, in Fluorination. Synthetic Organofluorine Chemistry, ed. J. Hu and T. Umemoto, Springer, Singapore, 2018

[cit21] Weiss H.-C., Bläser D., Boese R., Doughan B. M., Haley M. M. (1997). ChemComm.

[cit22] Hei Z. H., Song G. L., Zhao C.-Y., Fan W., Huang Mu-H. (2016). RSC Adv..

[cit23] Wachowski L., Sobczak J. W., Hofman M. (2007). Appl. Surf. Sci..

[cit24] Seredych M., Szczurek A., Fierro V., Celzard A., Bandosz T. J. (2016). ACS Catal..

[cit25] Nanse G., Papirer E., Fioux P., Moguet F., Tressaud A. (1997). Carbon.

[cit26] Wang X., Dai Y., Gao J., Huang J., Li B., Fan C., Yang J., Liu X. (2013). ACS Appl. Mater. Interfaces.

[cit27] Asanov I. P., Bulusheva L. G., Dubois M., Yudanov N. F., Alexeev A. V., Makarova T. L., Okotrub A. V. (2013). Carbon.

[cit28] Nair R. R., Ren W., Jalil R., Riaz I., Kravets V. G., Britnell L., Blake P., Schedin F., Mayorov A. S., Yuan S., Katsnelson M. I., Cheng H.-M., Strupinski W., Bulusheva L. G., Okotrub A. V., Grigorieva I. V., Grigorenko A. N., Novoselov K. S., Geim A. K. (2010). Small.

[cit29] Ferrari A., Basko D. M. (2013). Nat. Nanotechnol..

[cit30] Bernal J. D. (1924). Proc. R. Soc. Edinburgh.

[cit31] Caldas V., Morin F. G., Brown G. R. (1994). Magn. Reson. Chem..

[cit32] Giraudet J., Dubois M., Guerin K., Pinheiro J. P., Hamwi A., Stone W. E. E., Pirotte P., Masin F. (2005). J. Solid State Chem..

[cit33] Schwarz D., Noda Y., Klouda J., Schwarzová-Pecková K., Tarábek J., Rybácek J., Janoušek J., Simon F., Opanasenko M. V., Cejka J., Acharjya A., Schmidt J., Selve S., Reiter-Scherer V., Severin N., Rabe J. P., Ecorchard P., He J., Polozij M., Nachtigall P., Bojdys M. J. (2017). Adv. Mater..

[cit34] Yang Z., Wang N., He J., Wang K., Li X., Shen X., Wang X., Lv Q., Zhang M., Jiu T., Hou Z., Huang Ch. (2018). Carbon.

[cit35] https://gaussian.com/citation/

[cit36] Becke A. D. (1993). J. Chem. Phys..

[cit37] Weigend F., Ahlrichs R. (2005). Phys. Chem. Chem. Phys..

[cit38] Feng Z., Li Y., Tang Y., Chen W., Li R., Ma Y., Dai X. (2020). J. Mater. Sci..

[cit39] Jeon K.-J., Lee Z., Pollak E., Moreschini L., Bostwick § A., Park C.-M., Mendelsberg R., Radmilovic V., Kostecki R., Richardson T. J., Rotenberg E. (2011). ACS Nano.

[cit40] Samarakoon D. K., Chen Z., Nicolas C., Wang X.-Q. (2010). Small.

[cit41] Bulusheva L. G., Okotrub A. V., Boltalina O. V. (1999). J. Phys. Chem. A.

[cit42] Fedoseeva Y. V., Bulusheva L. G., Okotrub A. V., Vyalikh D. V., Fonseca A. (2010). J. Chem. Phys..

[cit43] Fijalkowski K. J., Jurczakowski R., Kożmiński W., Grochala W. (2012). Phys. Chem. Chem. Phys..

[cit44] Fijalkowski K. J. , Jurczakowski R., *PL Pat.*, 221643, 2016; *EU Pat.*, 2788745, 2016; *US Pat.*, 9651595, 2017; *JP Pat.*, 6219831, 2017

[cit45] An H., Li Y., Feng Y., Cao Y., Cao C., Long P., Li S., Feng W. (2018). ChemComm.

[cit46] Yang Z., Shen X., Wang N., He J., Li X., Wang X., Hou Z., Wang K., Gao J., Jiu T., Huang C. (2019). ACS Appl. Mater. Interfaces.

[cit47] Xing Z., Ju Z., Zhao Y., Wan J., Zhu Y., Qiang Y., Qian Y. (2016). Sci. Rep..

[cit48] Zhang S., He J., Zheng J., Huang C., Lv Q., Wang K., Wang N., Lan Z. (2017). J. Mater. Chem. A.

[cit49] Lee W. C., Lim C. H. Y. X., Shi H., Tang L. A. L., Wang Y., Lim C. T., Loh K. P. (2011). ACS Nano.

[cit50] Wang Y., Lee W. C., Manga K. K., Ang P. K., Lu J., Liu Y. P., Lim C. T., Loh K. P. (2012). Adv. Mater..

