# Supplementary material for: Toward the synthesis, fluorination and application of N–graphyne
Source: RSC Adv. 2020 Nov 3;10(66):40019–29. doi: 10.1039/d0ra08143d (PMC9057576; doi:10.1039/d0ra08143d)
Supplement: RA-010-D0RA08143D-s001 [file RA-010-D0RA08143D-s001.pdf]

Electronic Supplementary Material (ESI) for RSC Advances.
This journal is © The Royal Society of Chemistry 2020

ELECTRONIC SUPPLEMENTARY INFORMATION

Toward synthesis, fluorination and application of N-graphyne

Gisya Abdi[a], Anna Filip[†b], Michał Krajewski[†c], , Krzysztof Kazimierczuk[a], Karol J. Fijałkowski[a], Marcin Strawski[c], Paweł Szarek[a], Zoran Mazej[d], Grzegorz Cichowicz[e], Piotr J. Leszczyński[a], Andrzej Szczurek*[a] Wojciech Grochala[a]

[a] *Centre of New Technologies, University of Warsaw, Żwirki i Wigury 93, 02-097 Warsaw, Poland*

[b] *Laboratory of Molecular Medical Biochemistry, Nencki Institute of Experimental Biology, PAS, Pasteura 3, 02-093 Warsaw, Poland*

[c] *Faculty of Chemistry, University of Warsaw, Pasteura 1, 02-093 Warsaw, Poland*

[d] *Department of Inorganic Chemistry and Technology, Jozef Stefan Institute, Jamova 39, SI 1000 Ljubljana, Slovenia*

[e] *The Czochralski Laboratory of Advanced Crystal Engineering, Faculty of Chemistry, University of Warsaw, Żwirki i Wigury 101, 02-089 Warsaw, Poland*

MATERIALS AND METHODS

Materials. 1,3,5-tribromobenzene (1,3,5-TEB) (97%, Sigma Aldrich), Trimethylsilylacetylene (97%, Sigma Aldrich), Zinc Chloride (99,999%, ABCR), n-BuLi (2M in cyclohexane, Sigma Aldrich) Cyanuric chloride (99%, Sigma Aldrich), Pd(PPh3)4 (99%, Sigma Aldrich), solvents: THF, diethyl ether, DMF (all anhydrous, Sigma Aldrich). All chemicals were stored in dry conditions of the MBraun glove box.

Synthesis of N-Graphynes. In the first step, the ZnCl@trimethylsilylacetylene complex (10mmol) was prepared. For this purpose, a three-neck flask flushed with nitrogen flow was filled with1,43 ml of (trimethylsilyl)acetylene (10 mmol) and 4 ml of anhydrous THF. The flask was next cooled to –78°C. When the temperature reached an equilibrium, 6.3 ml of a hexane solution of N-butyllithium (1,56mol/L) was added dropwise over a period of 30 minutes. After adding all volume of n-buLi the mixture was stirred for additional 60 minutes at –78°C. Thereafter, the suspension of 1.43 g of ZnCl in 6 ml of THF was added to the mixture. After an additional 60 minutes of stirring at –78°C, the mixture was warmed slowly up to room temperature. In the second step 1,3,5-tris(trimethylsilyl)ethynylbenzene was prepared in the reaction of 1.259 g (4 mmol) dissolved in 6 ml of THF with previously prepared 10 mm of Zn complex in presence of 0.5 g of $Pd(PPh_3)_4$ in 20 ml of toluene used as a catalyst. The reaction was carried out in the protective atmosphere of argon at 60°C for 72 hours. After completing the reaction, 20 ml of 1N HCl was added, the mixture was extracted with diethyl ether (4x50 ml). The combined organic layer was next washed with brine (150 ml) and dried over $MgSO_4$. The obtained brown crude product was purified by column chromatography (Dichloromethane/ethyl acetate 10:1).

The protecting trimethylsilyl groups were removed in reaction with 1M TBAF at low temperature followed by three-time washing with saline solution, dried over $MgSO_4$ and the solvents were finally evaporated under vacuum. The obtained brownish solid was recrystallised by sublimation carried out in tubular furnace at 400°C and under nitrogen atmosphere. Obtained white needle crystals grew on the wall of the cold part of the quartz tube where collected and used in a cross-coupling reaction. These crystals were also investigated by monoc-rystalline XRD to confirm the purity and crystal structure of 1,3,5-Triethynylbenzene.

The cross-coupling reaction of received 1,3,5-triethynylbenzene and cyanuric chloride (molar ratio 1:1) was carried out for 24 h in a sealed 100 ml Teflon-lined stainless steel reactor and heated up to 120°C. The reaction was carried out in 30 ml dimethylformamide (DMF) and 1 ml trimethylamine (TEA) as a solvent and in the presence of $Pd(PPh_3)_4$ as a catalyst and CuCl (co-catalyst). Obtained dark brown/black gels were washed with methanol, ethanol, and diethyl ether, the inorganic traces were removed by 1M of HCl. Finally, N-Graphyne was washed with deionized water and dried in the oven at 105°C for 24h.

Fluorination of N-Graphynes. N-Graphyne was fluorinated with Xenon(II) fluoride in conditions allowing to obtain different fluorination rate. In this purpose different mass ratios N-Gy/XeF_2 1.5, 6.7, and 10 were applied. All the manipulations were carried out under anhydrous conditions. The volatile compounds, such as CH_2Cl_2, were handled on a vacuum line constructed of nickel-Teflon, and the non-volatile materials were handled in the MBraun glove box under an argon atmosphere (<0.5 ppm H_2O). Syntheses were carried out in FEP (tetrafluoroethylene-hexafluoropropylene block copolymer; Polytetra GmbH, Germany) reaction vessels (16 mm i.d. and 19 mm. o.d.) equipped with PTFE valves. Xenon difluoride was prepared by the photochemical reaction between Xe and F_2 at ambient temperature [S1]. Dichloromethane and xenon(II) fluoride were condensed to the solid at 77 K and the reaction mixture was first brought to -40°C. Since there was no visible reaction the reaction mixture was brought to ambient temperature. The reaction mixture was left stirring over 2-4 nights at ambient temperature. All volatiles were slowly pumped off at ambient temperature. Removed liquid CH_2Cl_2 was colorless. Final products' color was changing with used ratio and was red-brown for ratio 1.5 or yellow for ratios 6.7 and 10. TCC directly mixed with solid XeF2 (ratio 10) under ambient condition caused a very fast and violent reaction between reactants resulting in decomposition of the sample.

CHARACTERIZATION

Single crystal XRD of 1,3,5-triethynylbenzene was performed at 130(2) K on a Bruker D8 Venture Photon II diffractometer equipped with a TRIUMPH monochromator and a MoKα fine focus sealed tube (λ = 0.71073 Å). A total of 1608 frames were collected with Bruker APEX3 program [S2]. The frames were integrated with the Bruker SAINT software package [S3] using a narrow-frame algorithm. The integration of the data using a monoclinic unit cell yielded a total of 10486 reflections to a maximum ϑ angle of 25.37° (0.83 Å resolution), of which 1551 were independent (average redundancy 6.761, completeness = 99.8%, R_{int} = 4.18%, R_{sig} = 2.60%) and 1258 (81.11%) were greater than 2σ(F^2). The final cell constants of a = 19.0132(10) Å, b = 3.9430(2) Å, c = 23.6198(12) Å, β = 108.101(2)°, V = 1683.12(15) Å^3, are based upon the refinement of the XYZ-centroids of 5460 reflections above 20 σ(I) with 6.609° < 2θ < 50.51°. Data were corrected for absorption effects using the Multi-Scan method (SADABS) [S4]. The ratio of minimum to maximum apparent transmission was 0.840. The calculated minimum and maximum transmission coefficients (based on crystal size) are 0.9570 and 0.9870. The structure was solved and refined using the Bruker SHELXTL Software Package, using the space group *C*2/*c*, with Z = 8 for the formula unit, $C_{12}H_6$. The final anisotropic full-matrix least-squares refinement on F^2 with 110 variables converged at R_1 = 3.35%, for the observed data and wR_2 = 9.01% for all data. The goodness-of-fit was 1.039. The largest peak in the final difference electron density synthesis was 0.157 e$^-$/Å^3 and the largest hole was –0.123 e$^-$/Å^3 with an RMS deviation of 0.037 e$^-$/Å^3. On the basis of the final model, the calculated density was 1.185 g/cm^3 and F(000), 624 e$^-$. was –0.178 e$^-$/Å^3 with an RMS deviation of 0.069 e$^-$/Å^3. On the basis of the final model, the calculated density was 1.724 g/cm^3 and F(000), 624 e$^-$.

All carbon atoms were refined anisotropically. All of hydrogen atoms were placed in calculated positions and refined within the riding model. The temperature factors of all other hydrogen atoms were not refined and were set to be either 1.2 times larger than U_{eq} of the corresponding carbon atom. The atomic scattering factors were taken from the International Tables [S5]. Molecular graphics was prepared using program Mercury CSD 3.9 [S6]. Thermal ellipsoids parameters are presented at 50% probability level. Crystal data and the structure refinement parameters are presented in Table 1.

Powder XRD. X-ray diffraction (XRD) profiles were measured with a Panalytical X'Pert Pro diffractometer. The latter was used in a Bragg–Brentano configuration in reflection, and was equipped with a Co anticathode and a linear PIXel detector. The materials were ground in an agathe mortar and pistel, and the resultant powders were placed in quartz capillary tubes (fi = 0.5 mm). The latter was installed on a rotating spinner for allowing the highest number of grain to be in diffraction position.

Morphology and chemical composition. Fluorinated N-Graphyne samples were firstly investigated by transmission electron microscope (TALOS F200X) working with electron excitation energy of 200 keV. The energy-dispersive X-ray spectroscopy was conducted in the order to investigate the elemental characterization and dispersion of main elements being present on the sample surface. Prior the observations, powdered samples were ultrasonically dispersed in acetone and transferred onto 200 mesh carbon-coated copper grid (Electron Microscopy Science, USA).

Photoluminescence. The photoluminescence measurements were carried out in room temperature for ethanol suspensions of fluorinated materials using Horiba Fluorolog-3 spectrofluorometer. Samples were excited with excitation wavelength 380 nm generated by Xenon lamp.

FTIR. The chemical structure of fluorinated N-Graphynes was investigated in a vacuum using Vertex 80 spectrometer (Bruker, USA) over the 4000–400 cm^{-1} range, by averaging 50 scans at a maximum resolution of 1 cm^{-1}. For that purpose, pellets made from 1 mg of dried samples ground, and pressed with 100 mg of dry potassium bromide.

Raman. Raman spectra were measured on Horiba T64000 Raman spectrometer using pressed pieces of pristine and fluorinated N-Graphynes. The spectra were recorded under a microscope using a 20x·objective. A laser of wavelength 514 nm was used. Each spectrum was obtained by accumulation of 30 spectra recorded from 100 to 4000 cm^{-1} and an acquisition time was fixed at 10 s. Obtained spectra were fitted by using the Gaussian–Lorentzian profiles in the first-order region for the D and G bands.

^{13}C and ^{19}F MAS NMR. Solid-state NMR spectra were measured on an Agilent 700 MHz spectrometer at room temperature using 3.2 mm HXY triple-channel probe under MAS rate of 18 kHz. Standard-wall zirconia MAS rotors have been used. In ^{13}C NMR experiments we used a simple pulse-acquire sequence with the ^{13}C $\pi/2$ pulse length was 6.0 μs with a recycle delay of 4 s, 512 scans, spectral width of 65789 Hz and 100 ms acquisition time. Spectra were exponentially weighted with line-broadening of 40 Hz. In ^{19}F NMR experiments we used a simple pulse-acquire sequence with the ^{19}F $\pi/2$ pulse length was 4.0 μs with a recycle delay of 5 s, 256 scans, spectral width of 131578 Hz and 15.6 ms acquisition time. Spectra were exponentially weighted with line-broadening of 100 Hz.

XPS. X-ray photoelectron spectroscopy spectra were acquired using a Kratos Axis Supra spectrometer, equipped with a monochromatic Al Kα radiation (1486.7 eV) source. The instrument work function was calibrated to give a BE of 84.0 eV ± 0.1 eV for the 4f 7/2 line of metallic gold and the spectrometer dispersion was adjusted to give a BE of 932.62 eV for the Cu 2p 3/2 line of metallic copper. Energy resolution was examined on silver sample. Survey (wide) spectra were collected with a quality corresponding to the FWHM parameter for Ag 3d line equal to 0.71 eV at energy step size equal to 0.5 eV. For high resolution spectra the FWHM parameter for Ag 3d line was equal to 0.58 eV at step size was: 0.1 eV. Kratos charge neutralizer system was used to reduce charge compensation. The peaks fitting was conducted using CasaXPS software version 2.3.18 on a Shirley background. All spectra were calibrated using the adventitious C 1s peak with a fixed value of 285 eV.

EIS. Electrical impedance spectroscopy measurements were carried out using a Solartron 1260A Frequency Response Analyser equipped with 1296A dielectric interface and coupled with IMPED CELL sample holder. IMPED CELL is an in-house designed tool to investigate pristine powder samples under inert atmosphere in a function of temperature and pressure simultaneously. EIS spectra were collected within the frequency range of 10^{7}–10^{-2} Hz and AC amplitude of 10–1000 mV.

Computations. The computations were done using Gaussian 16 [S6] by hybrid functional B3LYP [S7] with Def2SVP basis set [S8]. The optical gap has been determined from the eigenvalues of HOMO and LUMO Kohn−Sham orbitals and is given as:

$$\Delta E_{HL} = E_{LUMO} - E_{HOMO}$$

The structures varying in fluorine content and type of CF functional groups taken for DFT computations are shown in Scheme 1 below.

Cytotoxicity test. In order to investigate the effect of pristine and fluorinated N-graohyne on HEK cells (HEK293 cell line, ACCT, Sigma-Aldrich, Poland) the cell viability test was performed using the CellTiter Blue Reagent (CTB, Promega, Poland). HEK cells were plated in the 96 well plate in a growth medium composed of RPMI (Biowest, VWR Poland) with 1% penicillin/streptomycin, 20 mM l-glutamine (provided by Sigma–Aldrich, Poland) containing 10% decomplemented FBS (Biowest, VWR Poland) until confluence, in a 37°C, 5% CO_2 humidified cell culture incubator. At confluence, HEK cells were treated with substance F1, F1, Ko, HCC or CCC suspended in growth medium at concentration of 300 µg/ml, 200 µg/ml, 100 ug/ml, 50 µg/ml, 40 µg/ml or 20 µg/ml for 24 hours. After 24 hours of incubation cells underwent CTB assay according to the manufacturer recomendation (Promega). The results obtained from three independent cell cultures were expressed as the difference between the light absorbance at 573 nm (converted dye) and the absorbance at 605 nm (background). These values were directly proportional to the number of living cells in the culture.

Antibacterial tests. Antibacterial tests were performed using Agar disk-diffusion method. The Petri dishes with agar were inoculated with a standardized inoculum of the tested microorganism, E. Coli. In next step, filter paper discs containing the studied compound at a desired concentration, varying from 12,5 to 100 µg were placed on the agar surface. That prepared Petri dishes were next incubated (in dark) overnight at 37°C. After time of incubation the growth area was assessed.

RESULTS

Scheme S1. N-graphyne structures considered in DFT computations.

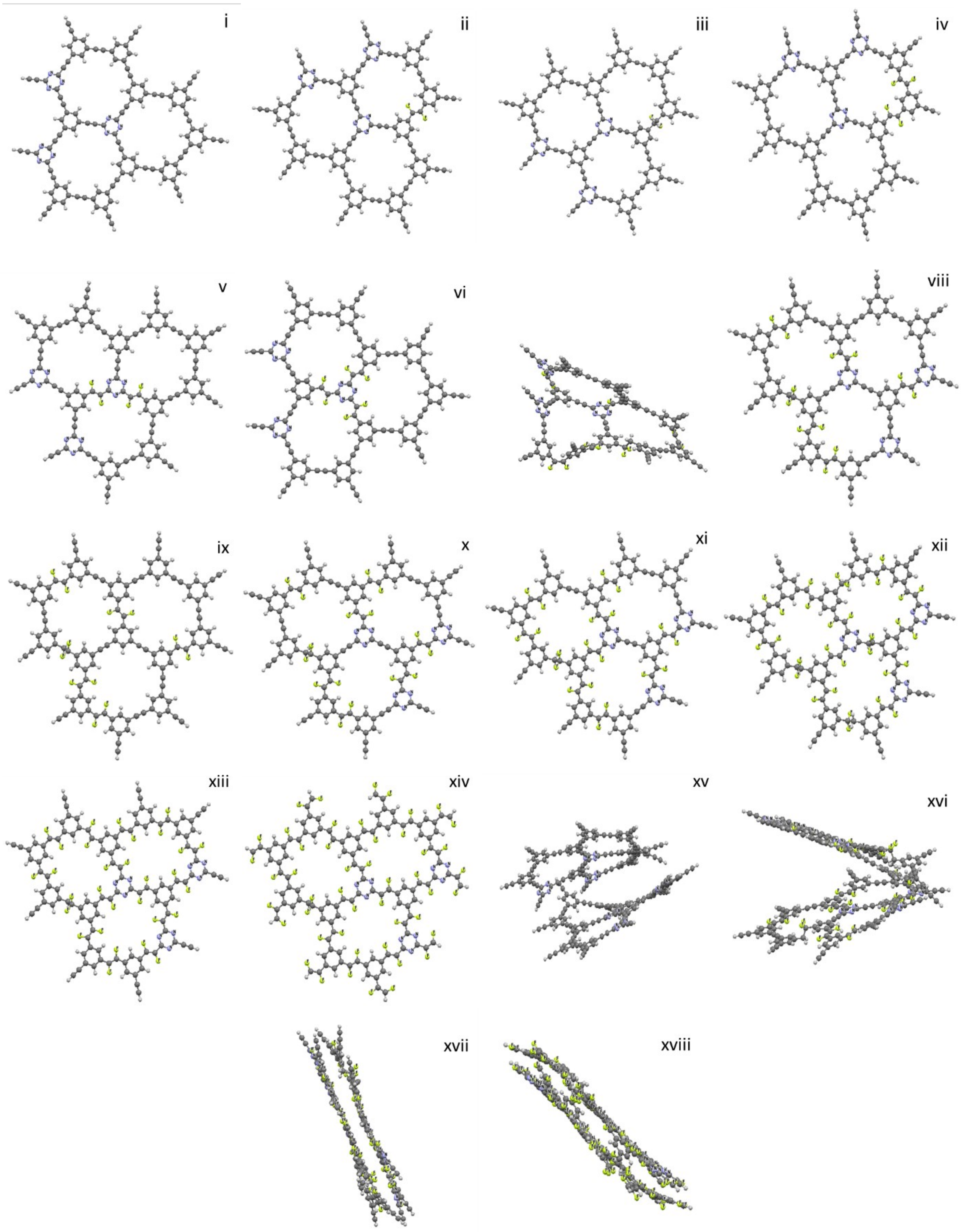

Figure S1. Crystals 1,3,5-TEB grown on the surface of reaction tube.

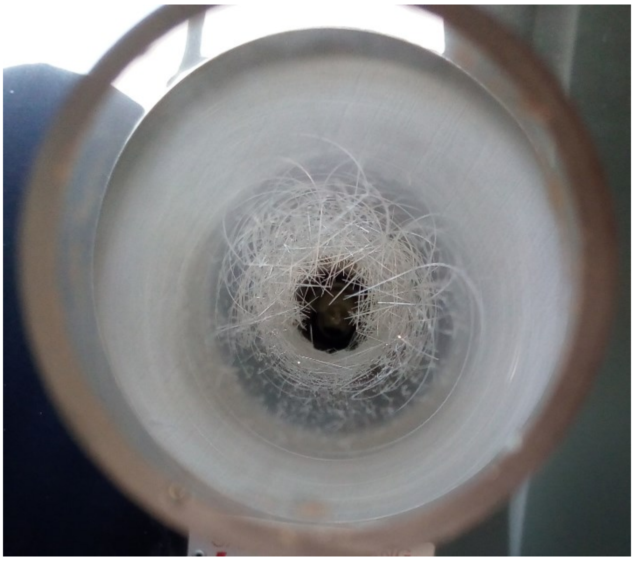

Figure S2. Packing diagrams of 1,3,5-TEB along [010] (A) and [100] (B) directions.

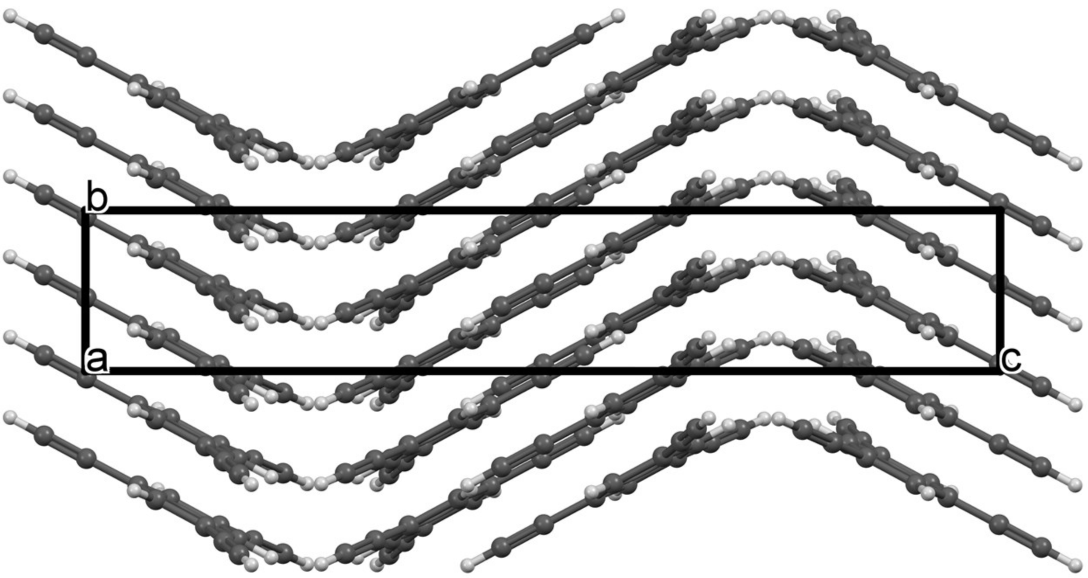

A

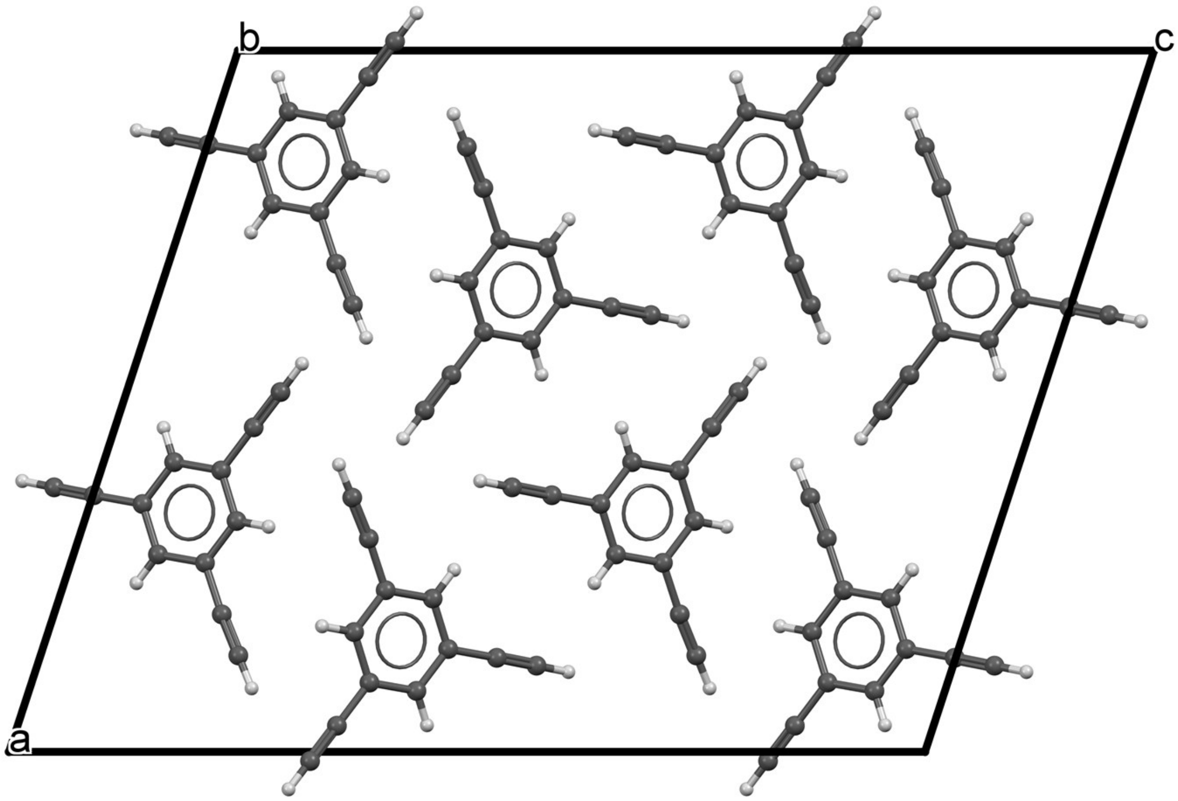

B

Table S1. Crystal data and structure refinement parameters for 1,3,5-TEB.

Indentification code	1,3,5-TEB
**Formula**	$C12H6$
**Ms/ g/mol**	150.17
**T/ K**	130(2)
**λ/ Å**	0.71073
**Crystal size/ mm**	0.194 x 0.211 x 0.657
**Crystal system**	Monoclinic
**Space group**	*C*2/*c*
**Unit cell dimensions**	*a* = 19.0132(10) Å<br>*b* = 3.9430(2) Å<br>*c* = 23.6198(12) Å<br>*β* = 108.101(2)°
**V/ Å^3, Z**	1683.12(15), 8
**D/ g/cm^3**	1.185
**μ/ mm^{-1}**	0.067
**F (000)**	624
**ϑ_{min}, ϑ_{max}**	3.30°, 25.37°
**Index ranges**	-22 ≤ h ≤ 22,<br>-4 ≤ k ≤ 4,<br>-28 ≤ l ≤ 28
**Reflections collected**	10486
**Independent reflections**	1551 [R_{int} = 0.0418]
**Completeness**	99.8%
**Absorption correction**	multi-scan
**T_{max}, T_{min}**	0.9870, 0.9570
**Data / restraints / parameters**	1551 / 0 / 110
**Goodness-of-fit on F^2**	1.039
**Final *R* indices**	1258 data; I > 2σ(I)<br>R_1 = 0.0335, wR_2 = 0.0815<br>all data<br>R_1 = 0.0479, wR_2 = 0.0901
**Extinction coefficient**	0.0062(12)
**ρ_{max}, ρ_{min} / e·Å^3**	0.157, -0.123

Figure S3. Fluorination of TCC. The solution of TCC in dichloromethane (A), starting reaction mixture of TCC with XeF_2 in CH_2Cl_2 (B), dry fluorinated product (TCCF3) (C).

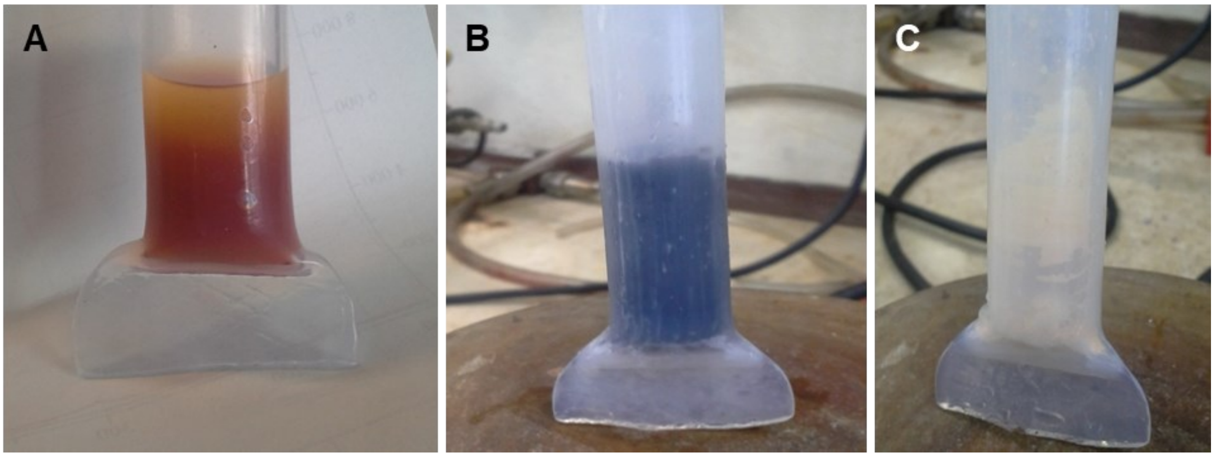

Figure S4. Set of TEM micrographs of fluorinated N-Graphynes TCCF1 sample, BF) bright field overall view. Images of EDX elemental distribution showing content of: carbon (C), oxygen (O), and fluorine (F).

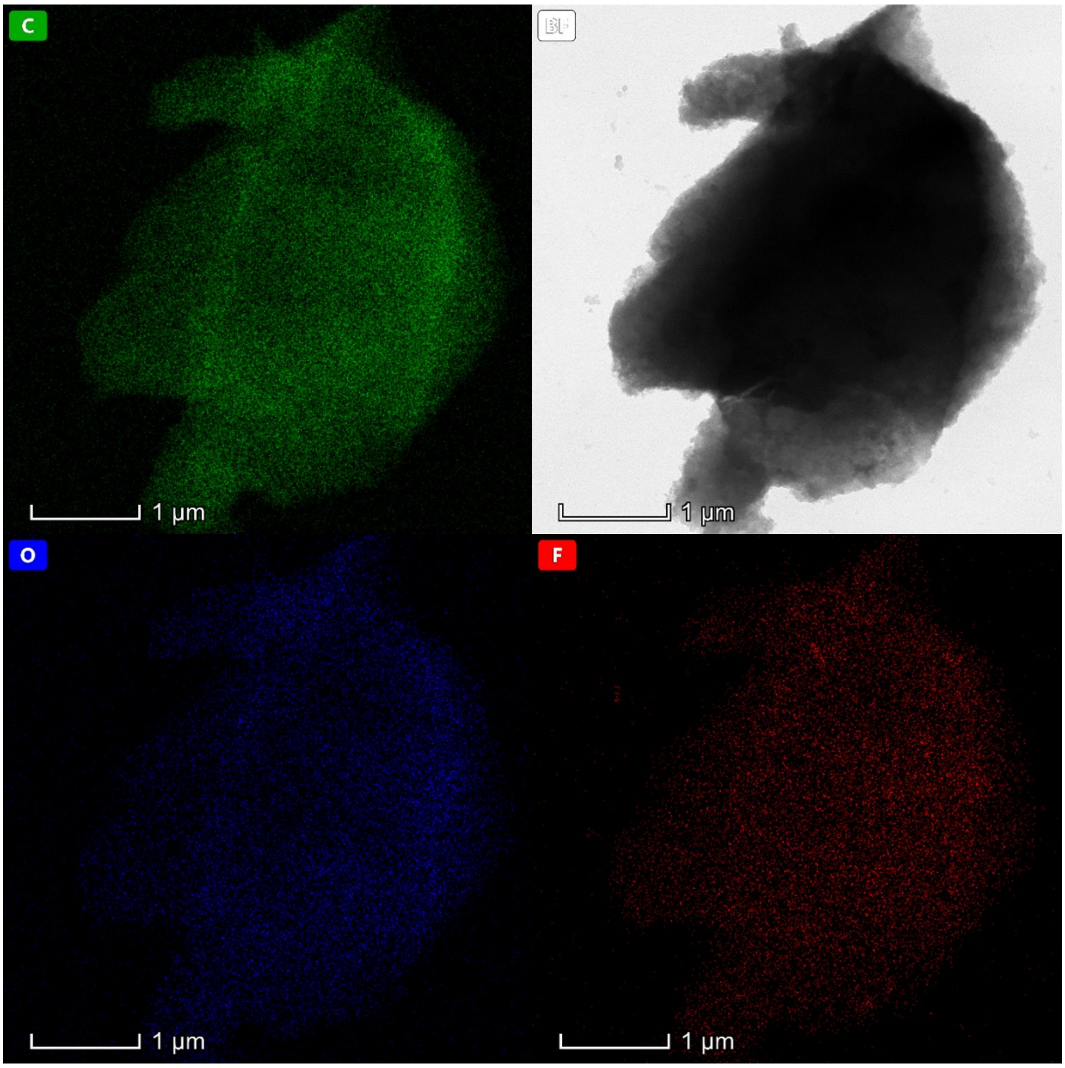

Table S2. C1s XPS deconvolution data. Location, ascription and amount of chemical species in TCC and TCCFs samples.

Functional groups	TCC eV/at%	TCCF1 eV/at%	TCCF2 eV/at%	TCCF3 eV/at%
**C=C**	284.4 / 63.7	284.3 / 48.5	284.4 / 36.9	284,9 / 31.9
**C-C (sp)**	284.9 / 15.2	285.3 / 9.6	284.9/8.5	284.9/2.9
**C-O**	285.7 / 5.4	285.5 / 11.4	285.5 / 14.8	286,4 / 11.7
**C-N**	286.8/9.1	286.3/10.3	286.3/7.1	286.4/5.0
**C=N, C=O**	287.8 / 6.8	287.3 / 7.8	287.4 / 10.0	287.5 / 16,6
**Csp_2-F**	-	288.5 / 7.8	288.4 / 10.7	288.3 / 14,4
**Csp_3-F**	-	289.4/3.5	289.6 / 7.8	289.5 / 9.6
CH_2-CF_2	-	291.1/1.1	291.0 / 3.0	291,8 / 4.7
**CF_2-CF_2**	-	-	292.4/1.2	292.4/3.3

Table S3. F1s XPS deconvolution data. Location, ascription and amount of chemical species in TCCFs samples.

Functional groups	TCCF1 eV / at%	TCCF2 eV / at%	TCCF3 eV / at%
**isolated CF**	684.8 / 22,7	684,8/ 9.5	-
**CF=CF**	286,3 / 66.2	686,9/74,5	686.9 / 80.0
**CH-CF_2, CF_2-CF**	288,1 / 11.7	688.0/13.5	688.0 / 9.1
**CF_2-CF_2**	-	689.1 / 2,4	689.9 / 10,9

Figure S5. Exemplary Raman spectroscopy of TCCF2 sample. The same appearance of Raman spectra was observed for all fluorinated samples.

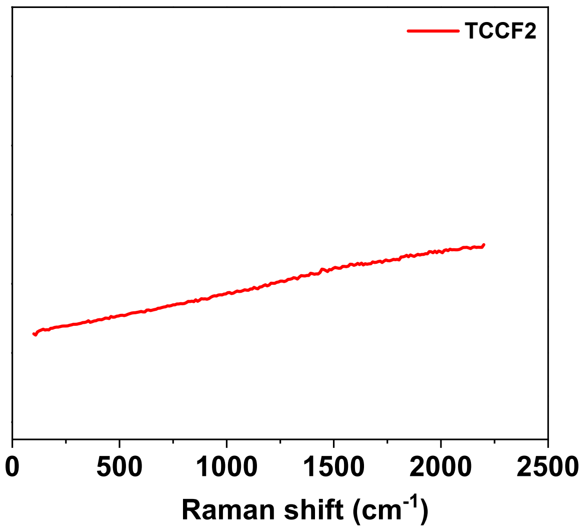

Figure S6. 19F solid state NMR of TCCF3 sample.

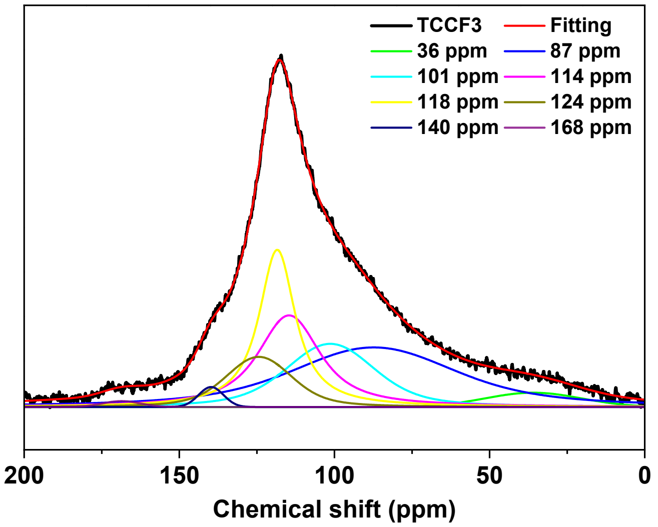

Figure S7. The PL spectra of fluorinated samples measured at room temperature at the excitation wavelength of 380 nm. Inset picture

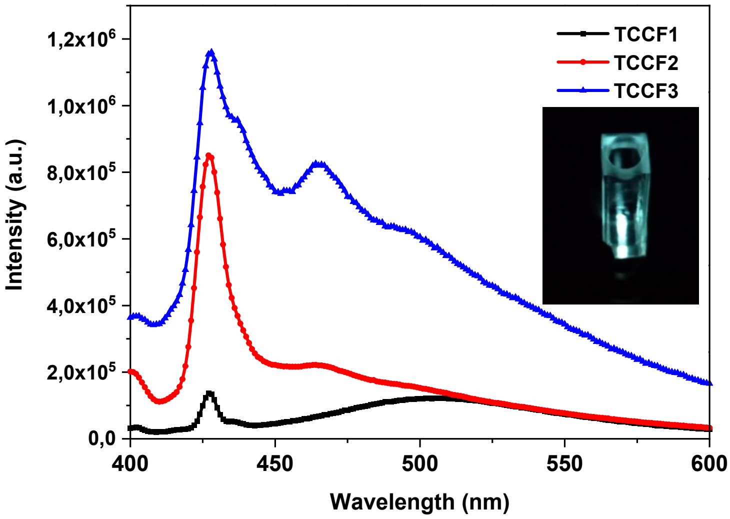

Investigated samples, whatever the fluorine content, were characterized by spectra with a broadband emission profile varying from 420 to 550 nm. Such broad spectra are characteristic of amorphous semiconductors with broad tails mostly due to structural disorders. The characteristic band at 427 nm, found for all investigated samples was probably a Raman peak of the solvent and shouldn't be considered in spectra identification. The distinct blue emission shift in a function of fluorine concentration was observed for peaks seen at 504 nm (2,457eV) for TCCF1 through 467 nm (2,655eV) to 463 nm (2,677 eV) for samples TCCF2 and TCCF3 respectively. In the case of the TCC1 sample, one broad peak was observed, whereas additional peaks at 496 nm (2,498 eV) and 489 nm (2,538 eV) appeared for samples TCCF2 and TCC3, respectively. The separation of the letter peaks was equal to 0.139 eV for TCCF3 and 0.150eV for TCCF2 what directly corresponds to vibrational mods at 1121 and 1209 cm^{-1} found for CF units presented already in figure 3A. The origin of blue emission of Fluorinated N-Graphynes lies in the formation of isolated sp^2 clusters during the fluorination step. These clusters are built from aromatic substituents - including heteroaromatic triazine and -CF=CF- linkers containing Csp^2-F units. Moreover, the possibility of coexistence of two different settings of fluorine atoms in space, and thus two structural E and Z isomers of fluoroaliphatic linkers, increases the number of available modes of chemical bond vibration in the cluster structure. Increasing the number of available modes of vibration of chemical bonds in the structure of clusters and strong coupling of π electrons between aromatic substituents allows the occurrence of fluorescence.

Table S4. HOMO-LUMO gap values calculated for different arrangements of fluorinated N-gtraphynes.

Comment	# of F	# of C≡C	HOMO	LUMO	Eopt = EL - EH
**i) unsubstituted**	**0**	**24**	**-6,3139**	**-2,8803**	**3,4336**
**ii) trans-FC=CF**	2	23	-6,3098	-2,8757	3,4341
**iii) CH2CF2**	2	23	-6,2608	-2,8978	3,3631
**iv) trans-FC=CF around benzene ring**	4	22	-6,3093	-2,8735	3,4357
**v) trans around central triazine ring**	4	22	-6,3128	-2,9576	3,3552
**vi) trans around central triazine ring**	6	21	-6,2992	-2,9527	3,3465
**vii) deformed - clamed structure**	12	18	-6,3321	-2,9816	3,3506
**viii) 5x trans FC=CF, 1x CH2CF2**	12	18	-6,2364	-2,9421	3,2942
**ix) 5x trans FC=CF, 1x CH2CF2; structure without triazine rings**	12	18	-6,2034	-2,4180	3,7854
**x) 7x trans FC=CF, 1x CH2CF2**	16	17	-6,2271	-2,9394	3,2877
**xi) 9x trans FC=CF, 1x CH2CF2**	20	14	-6,2053	-2,9337	3,2717
**xii) 11x trans FC=CF, 4x CH2CF2**	30	9	-6,2807	-3,1576	3,1231
**xiii) 14x trans FC=CF, 1x CH2CF2**	30	9	-6,2040	-2,9603	3,2436
**xiv) 23x trans FC=CF, 1x CH2CF2**	48	0	-6,1836	-2,9625	3,2210
**xv) 2x0**			-6,2059	-2,8727	3,3332
**xvi) 2x16**			-6,1068	-2,9296	3,1772
**xvii) 2x30**			-6,0908	-2,9595	3,1312

Based on structures ii), iii), xii) and xiii) the HL gaps seem to decrease with greater participation of CH_2CF_2 groups in the structure. The F substitutions near triazine rings decrease gaps compared to near-benzene ring substitutions, iv) and v) or viii) and ix). Deformed structures have higher gaps, vii) and viii). Comparing i), viii), x), xi), xiii) and xiv) the decrease in HL gaps might be observed with F saturation. Generally F-substituted structures have higher HOMO and lower LUMO than unsubstituted.

Figure S8. Real part of the permittivity for TCCF2 as a function of frequency, for various temperatures.

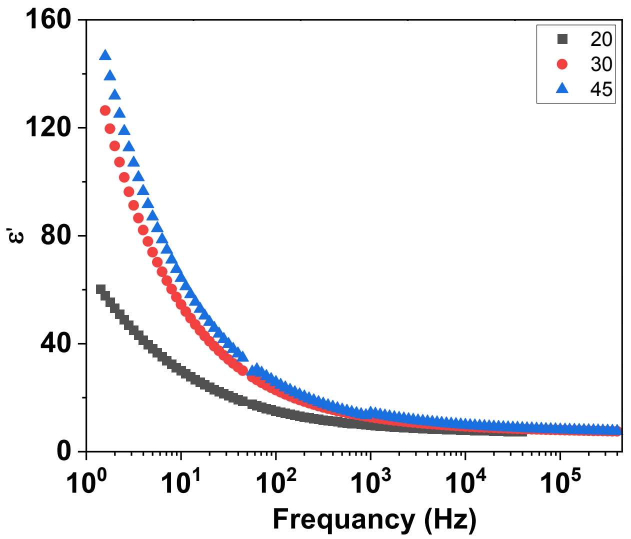

Figure S9. Antibacterial activity test conducted on TCCF1(a) and TCCF2 (b). The inset picture shows the average mass of material incorporated on a support.

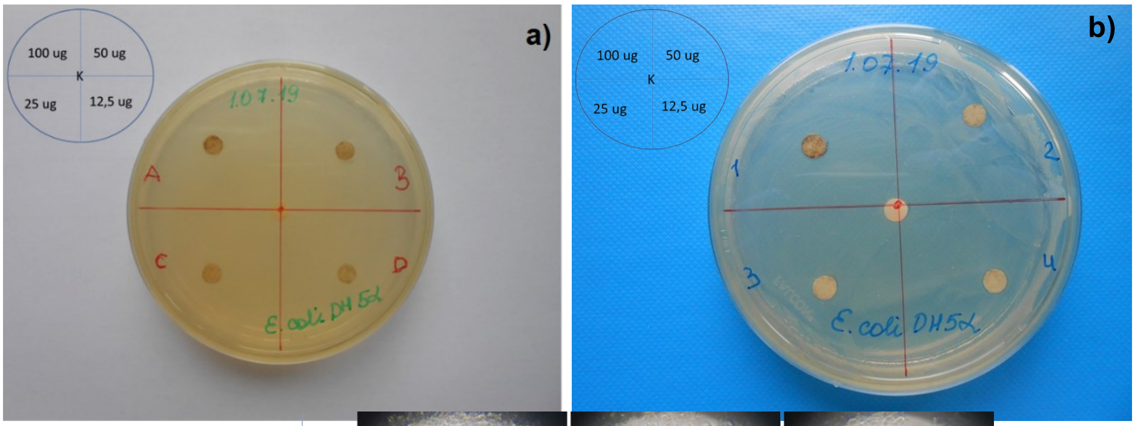

Figure S10. Pictures of grow[illegible] Pictures show the cell growth on pristine a[illegible]

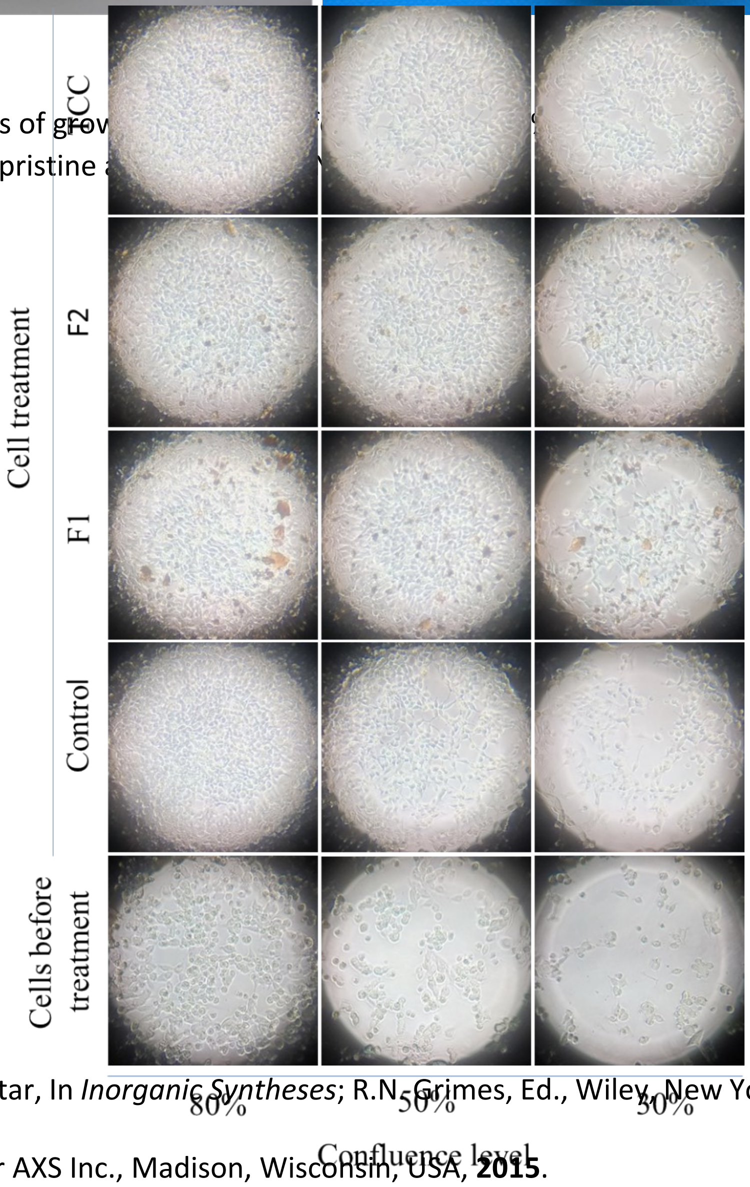

REFERENCES

S1. A. Šmalc, K. Lutar, In *Inorganic Syntheses*; R.N. Grimes, Ed., Wiley, New York, **1992**, Vol. 29, p. 1.

S2. APEX3,. Bruker AXS Inc., Madison, Wisconsin, USA, **2015**.

S3. SAINT,. Bruker AXS Inc., Madison, Wisconsin, USA, **2015**.

S4. SADABS,. Bruker AXS Inc., Madison, Wisconsin, USA, **2012**.

S5. *International Tables for Crystallography*, Ed. A. J. C. Wilson, Kluwer: Dordrecht, **1992**, Vol.C.

S6. C. F. Macrae, I. Sovago, S. J. Cottrell, P. T. A. Galek, P. McCabe, E. Pidcock, M. Platings, G. P. Shields, J. S. Stevens, M. Towler and P. A. Wood, *J. Appl. Cryst*., 2020, **53**, 226-235.
S7. https://gaussian.com/citation/
S8. A. D. Becke, *J. Chem. Phys.*, 1993, **98**, 5648.
S9. F. Weigend, R. Ahlrichs, Phys. Chem. Chem. Phys., 2005, **7**, 3297.
